# Inactivation of pentraxin 3 suppresses M2-like macrophage activity and immunosuppression in colon cancer

**DOI:** 10.1186/s12929-023-00991-7

**Published:** 2024-01-20

**Authors:** Feng-Wei Chen, Yung-Ling Wu, Chao-Chun Cheng, Yu-Wei Hsiao, Jhih-Ying Chi, Liang-Yi Hung, Chih-Peng Chang, Ming-Derg Lai, Ju-Ming Wang

**Affiliations:** 1 Institute of Basic Medical Sciences, College of Medicine, National Cheng Kung University, Tainan, Taiwan; 2 Department of Biotechnology and Bioindustry Sciences, College of Bioscience and Biotechnology, National Cheng Kung University, No. 1 University Rd., Tainan, 70101 Taiwan; 3 Department of Microbiology & Immunology, College of Medicine, National Cheng Kung University, Tainan, Taiwan; 4 Department of Biochemistry and Molecular Biology, College of Medicine, National Cheng Kung University, No. 1 University Rd., Tainan, 70101 Taiwan; 5 Graduate Institute of Medical Sciences, College of Medicine, Taipei Medical University, Taipei, Taiwan; 6 International Research Center for Wound Repair and Regeneration, National Cheng Kung University, Tainan, Taiwan; 7 Graduate Institute of Medicine, College of Medicine, Kaohsiung Medical University, Kaohsiung, Taiwan

**Keywords:** PTX3, Cancer-associated fibroblasts, M2-like macrophages, Colon cancer, Immunosuppression

## Abstract

**Background:**

The tumor microenvironment is characterized by inflammation-like and immunosuppression situations. Although cancer-associated fibroblasts (CAFs) are among the major stromal cell types in various solid cancers, including colon cancer, the interactions between CAFs and immune cells remains largely uncharacterized. Pentraxin 3 (PTX3) is responsive to proinflammatory cytokines and modulates immunity and tissue remodeling, but its involvement in tumor progression appears to be context-dependent and is unclear.

**Methods:**

Open-access databases were utilized to examine the association of PTX3 expression and the fibroblast signature in colon cancer. Loss-of-function assays, including studies in tamoxifen-induced *Ptx3* knockout mice and treatment with an anti-PTX3 neutralizing antibody (WHC-001), were conducted to assess the involvement of PTX3 in colon cancer progression as well as its immunosuppressive effect. Finally, bioinformatic analyses and in vitro assays were performed to reveal the downstream effectors and decipher the involvement of the CREB1/CEBPB axis in response to PTX3 and PTX3-induced promotion of M2 macrophage polarization.

**Results:**

Clinically, higher PTX3 expression was positively correlated with fibroblasts and inflammatory response signatures and associated with a poor survival outcome in colon cancer patients. Blockade of PTX3 significantly reduced stromal cell-mediated tumor development. The decrease of the M2 macrophage population and an increase of the cytotoxic CD8^+^ T-cell population were observed following PTX3 inactivation in allografted colon tumors. We further revealed that activation of cyclic AMP-responsive element-binding protein 1 (CREB1) mediated the PTX3-induced promotion of M2 macrophage polarization.

**Conclusions:**

PTX3 contributes to stromal cell-mediated protumor immunity by increasing M2-like macrophage polarization, and inhibition of PTX3 with WHC-001 is a potential therapeutic strategy for colon cancer.

**Supplementary Information:**

The online version contains supplementary material available at 10.1186/s12929-023-00991-7.

## Background

The tumor microenvironment, composed of diverse cell types and cytokines, plays a pivotal role in cancer progression and malignancy. Targeting tumor-associated stromal cells has emerged as a promising strategy to enhance the effects of anticancer treatment [1]. Cancer-associated fibroblasts (CAFs) constitute a substantial portion of the stromal cell population in colon cancer, and their presence is linked to an unfavorable survival prognosis. These CAFs are known to promote the development of malignant traits, resistance to therapy, self-activation, the suppression of antitumor immunity, and the amplification of immunosuppression [2].

Myeloid cells form a significant immune cell population within the tumor microenvironment and frequently adopt an immunosuppressive phenotype, thereby fostering malignancy or enabling immune evasion through the suppression of antitumor immunity [3, 4]. The tumor microenvironment contains a range of myeloid cell types, including myeloid-derived suppressor cells (MDSCs), tumor-associated neutrophils (TANs), and tumor-associated macrophages (TAMs). TAMs are typically categorized into two primary subtypes: proinflammatory M1 macrophages and anti-inflammatory M2 macrophages [5]. M2 macrophages can promote tumor growth and angiogenesis, or suppress cytotoxic T-cell activation and proliferation, leading to immunosuppression [6].

M2 macrophage polarization is triggered predominantly by anti-inflammatory cytokines such as IL-13 and IL-4 [7], as well as by other cytokines produced by tumor cells and stromal fibroblasts [8]. Moreover, following cytokine stimulation, multiple signaling pathways become activated, contributing to M2 macrophage polarization [9]. Cyclic AMP-responsive element-binding protein 1 (CREB1) is a phosphorylation-dependent transcription factor that drives the expression of genes involved in diverse cellular processes [10, 11]. CCAAT/enhancer-binding protein beta (CEBPB) is an important transcription factor that regulates the functions of monocyte lineage cells [12]. Activation of the CREB1/CEBPB axis can promote the expression of M2 macrophage markers [13], and the precise identification of crucial factors governing CREB1/CEBPB axis-mediated M2 macrophage regulation within the tumor microenvironment remains elusive.

Understanding the communication between colon cancer cells and stromal cells, including immune cells, is a crucial need for the development of anticancer drugs. Pentraxin 3 (PTX3), also known as TSG-14, is a member of a superfamily of highly conserved secretory proteins distinguished by a “pentraxin signature” [14]. PTX3 is expressed in various cell types and functions as a regulator of innate immunity and tissue remodeling [15, 16]. PTX3 expression is rapidly induced by proinflammatory cytokines, such as TNF-α and IL-1β [17], and PTX3 has thus been suggested to be a biomarker in inflammation-related diseases such as chronic obstructive pulmonary disease (COPD) [18], hepatocellular carcinoma [19] and liver fibrosis [20]. However, the role of PTX3 in cancer progression is debated and seems context dependent [21–28]. Our previous study showed that disruption of the PTX3/CD44 interaction attenuates breast cancer progression [29]. These opposing findings imply that the role of PTX3 may depend on the cancer type or the state of the tumor microenvironment. Several studies have demonstrated that PTX3 contributes to an anti-inflammatory phenotype in macrophages [30, 31], suggesting a potential function of PTX3 in the crosstalk among tumor cells, tumor-associated stromal cells, and tumor-associated macrophages.

In our study, we observed predominant expression of PTX3 in stromal cells within the colon tumor microenvironment. Inhibiting PTX3 led to a reduction in colon tumor growth in vivo, which was correlated with a decrease in M2-like macrophage infiltration and an increase in cytotoxic CD8^+^ T-cell infiltration. Furthermore, we demonstrated that PTX3 can induce the activation of the CREB1/CEBPB axis, promoting M2 macrophage polarization.

## Methods

### PTX3 neutralizing antibody/recombinant proteins

The monoclonal PTX3 antibody (WHC-001) and isotype IgG1κ used for in vitro and in vivo experiments were previously developed by our laboratory [29]. Briefly, the design and production of the anti-PTX3 neutralizing antibody were performed in cooperation with Leadgene Biomedical, Inc. (Tainan, Taiwan). Recombinant human wild-type and N220A PTX3 proteins were purified from Expi293 cells according to the manufacturer's instructions. Briefly, wild-type or N220A PTX3 expression plasmids were transfected into Expi293 cells by using the ExpiFectamine™ 293 Transfection Kit (Life Technologies Corporation, 5781 Van Allen Way, PO Box 6482, Carlsbad, CA 92008), and the supernatants were harvested for His-tag purification. Other recombinant proteins used in this study included murine PTX3 (#2166-TS-025/CF, R&D Systems, Inc., a Bio-Techne Brand, 614 McKinley Place NE, Minneapolis, MN 55413, USA), murine IL-1β (#211-11B, PeproTech, Part of Thermo Fisher Scientific, 5 Cedarbrook Drive, Cranbury, NJ 08512), murine TNFα (#ab259411, Abcam Inc., 1 Kendall Square, Ste 341, Cambridge, MA 02139-1517, USA), murine M-CSF (#576404, BioLegend Inc., 8999 BioLegend Way, San Diego, CA 92121, USA), murine IL-4 (#214-14, PeproTech, Part of Thermo Fisher Scientific, 5 Cedarbrook Drive, Cranbury, NJ 08512), murine IL-13 (#210-13, PeproTech), human IL-1β (#200-01B, PeproTech), and human TNFα (#ab9642, Abcam Inc., 1 Kendall Square, Ste 341, Cambridge, MA 02139-1517, USA).

### RNA sequencing analysis

Total cell lysates were homogenized in TRIzol reagent (Invitrogen Life Technologies, Carlsbad, California, USA), and the samples were sent to Biotools Co., Ltd. (Taiwan) for RNA sequencing analysis. The DNA library was constructed by Illumina paired-end sequencing. Briefly, quality control was performed by using FastQC, MultiQC, and ReSeqTools, and the raw reads were trimmed with Trimmomatic (v.0.38) to remove low-quality reads and adaptors. The DNA library was mapped to a reference genome (GRCh38, *Homo sapiens*) by using HISAT2. The read numbers were analyzed by featureCounts (v1.6.3) and normalized to TPM (transcript per million) values.

### Animal model

Female C57BL/6, BALB/c and NOD-SCID mice were purchased from the Laboratory Animal Center of National Cheng Kung University (Tainan, Taiwan) or BioLASCO (Taiwan Co., Ltd). Female *Ptx3* conditional knockout mice (C57BL/6J—*Ptx3*^*fl/fl*^ conditional knockout × B6.Cg-Ndor1^*Tg(UBC−Cre/ERT2)1Ejb/*^2 J mice) were bred and maintained in the National Applied Research Laboratory (Taiwan). All animal experiments were approved by the Institutional Animal Care and Use Committee (IACUC) at NCKU (IACUC No. 109037). C57BL/6 and BALB/c mice were 8–9 weeks old, transgenic mice (*Ptx3*^*fl/fl*^ and *Ptx3*^*fl/fl*^; Ubc-Cre) were 8–10 weeks old, and NOD-SCID mice were 6–7 weeks old when the experiments were performed.

For the mixed stroma-tumor (MEFs-MC38 cells) model, 8–9-week-old female C57BL/6 mice were anesthetized and 5 × 10^4^ MC38 cells mixed with 1 × 10^5^ MEFs were injected subcutaneously into the dorsal region (day 0); when the tumor volume reached 80–200 mm^3^, IgG1κ or WHC-001 (αPTX3 Ab) was intratumorally injected (100 μg in 100 μl) every 3 days for a total of 4 treatments by using 31G insulin syringes (BD Ultra-Fine 0.3 ml; Becton, Dickenson and Co.), and the mice were sacrificed on the 21 day post cell inoculation.

For the MC38 and CT26 cell subcutaneous tumor model, 8–9-week-old female C57BL/6 mice or BALB/c mice were anesthetized and 2 × 10^5^ MC38 or CT26 cells were injected subcutaneously in the dorsal region (day 0) and on the 8th day post cell inoculation, IgG1κ or WHC-001 (αPTX3 Ab) was intraperitoneally injected (10 mg/kg) every 4 days for a total of 3 treatments, and the mice were sacrificed on the 22nd day (MC38) or 20th day (CT26); 6–7-week-old female NOD-SCID mice were anesthetized and 2 × 10^5^ MC38 cells were injected subcutaneously in the dorsal region (day 0) and beginning on the 7th day post cell inoculation, IgG1κ or WHC-001 (αPTX3 Ab) was intratumorally injected (10 mg/kg) every 4 days for a total of 3 treatments, and the mice were sacrificed on the 21st day.

For the MC38 orthotopic tumor model, 8-week-old female C57BL/6 mice were anesthetized and 1 × 10^6^ MC38 cells in 30 μl of PBS were injected into the cecal wall (day 0) by using a 30G insulin syringe (BD Ultra-Fine 0.5 ml; Becton, Dickenson and Co.); beginning on the 7th day post cell inoculation, IgG1κ or WHC-001 (αPTX3 Ab) was intraperitoneally injected (10 mg/kg) every 4 days for a total of 3 treatments, and the mice were sacrificed on the 22nd day.

For the MC38 subcutaneous tumor model in *Ptx3* conditional knockout mice (C57BL/6 J—*Ptx3*^*fl/fl*^ conditional knockout × B6.Cg-Ndor1^*Tg(UBC−Cre/ERT2)1Ejb/*^2 J mice), the genotypes of the wild-type and knockout mice were *Ptx3*^*fl/fl*^ and *Ptx3*^*fl/fl*^;Ubc-Cre, respectively. For the latter knockout model, 8–10-week-old female or male mice were anesthetized and 2 × 10^5^ MC38 cells were injected subcutaneously into the dorsal region (day 0); beginning on the 5th day post cell inoculation, tamoxifen (2 mg/mouse in 10% EtOH corn oil) was intraperitoneally injected daily for a total of 5 days, and the mice were sacrificed on the 19th day. Tumor volume (mm^3^) = (L × W^2^)/2.

### Flow cytometric analysis

Tumor tissues were cut into small pieces and incubated in 5 ml digestion medium (RPMI-1640 medium containing 5% FBS, 1% P/S, 150 μl 10,000 CDU/ml collagenase I #C0103) at 37 °C on a shaker at 100 rpm for 40 min. A GentleMACS dissociator was used to homogenize the tissues into a single-cell suspension according to the manufacturer’s instructions. The suspension was filtered through a cell strainer (70 μm mesh). Tumor-infiltrating immune cells were stained with fluorescence-conjugated antibodies, including CD45-BV510 (#563891, BD), CD8a-PE (#553033, BD), CD4-BV421(#562891, BD), NK1.1-APC (#550627, BD), CD3e-PECy7 (#552774, BD), CD11b-BV421 (#562605, BD), Ly6G-FITC (#551460, BD), Ly6C-PECy7 (#560593, BD), F4/80-PE (#565410, BD), CD206-Alexa647 (#565250, BD), CD86-BB700 (#742120, BD), CD4-FITC (#553046, BD), and IFNγ-BV421 (#563376, BD). The populations were analyzed by using a CytoFLEX^™^ Flow Cytometer (Beckman Coulter). The CD45-positive immune cells were gated for CD8^+^ T cells (CD8^+^), CD4^+^ T cells (CD4^+^), NK cells (NK1.1^+^CD3e^−^), PMN-MDSCs (Ly6G^+^Ly6C^Mid^/CD11b^+^), M-MDSCs (Ly6C^+^Ly6C^low^/CD11b^+^), M1-like macrophages (CD11b^+^F4/80^+^CD206^−^ or CD11b^+^F4/80^+^CD86^+^CD206^−^), and M2-like macrophages (CD11b^+^F4/80^+^CD206^+^ or CD11b^+^F4/80^+^CD206^+^CD86^−^). For analysis of cytotoxic CD8^+^ T (IFNγ^+^CD8^+^/CD45^+^) and CD4^+^ T (IFNγ^+^CD4^+^/CD45^+^) cells, cells in suspension were further activated by incubation with stimulation medium (1.33 μM ionomycin, 20 ng/ml PMA, 10 μg/ml brefeldin A) at 37 °C for 4 h. Cells were harvested, and surface markers were stained with fluorophore-conjugated antibodies (CD45-BV510, CD8-PE, CD4-FITC). Stained cells were then fixed with 3.7% paraformaldehyde and stored at 4 °C overnight. The cells were permeabilized with Perm/Wash Buffer (#554723, BD BioSciences) and stained with the IFNγ-BV421 antibody for flow cytometric analysis.

### Bone marrow-derived macrophage differentiation

For experiments with *Ptx3* conditional knockout mice, 8–10-week-old female mice were intraperitoneally injected with tamoxifen (2 mg/mouse in 10% EtOH corn oil) daily for a total of 5 days (days − 5 to − 1), and the mice were sacrificed on the 8th day. The femurs and tibias were sterilized with 70% EtOH and washed with PBS. Bone marrow cells were flushed out with serum-free RPMI-1640 medium and collected. After centrifugation, red blood cells were removed by using Hybri-Max^™^ Red Blood Cell Lysing Buffer (R7757-100ML, Sigma) according to the manufacturer’s instructions. The cells were then filtered through a 70 μm mesh, washed with PBS and suspended in complete RPMI-1640 medium. For macrophage differentiation, a total of 4 × 10^6^ cells were seeded in 6-well plates with 2 ml complete RPMI-1640 medium containing 25 ng/ml M-CSF and incubated for 5 days (the medium and M-CSF were refreshed after 3 days). To induce M2 macrophage polarization, cells were treated with 20 ng/ml IL-4 and IL-13 for another 2 days, and RNA was harvested for analysis. For experiments with wild-type C57BL/6 mice, 8–10-week-old female mice were sacrificed, and the bone marrow cells were harvested as mentioned above. A total of 4 × 10^6^ cells were seeded in 6-well plates with 2 ml complete RPMI-1640 medium containing 25 ng/ml M-CSF and incubated for 7 days for macrophage differentiation (the medium and M-CSF were refreshed after 3 days). Macrophages were then cultured in medium without M-CSF for 24 h and treated with 100 ng/ml recombinant murine PTX3 protein for another 24 h.

For the experiment of BMDMs co-cultured with MEFs, 3 × 10^5^ MEFs (shVoid, shPtx3#915, and shPtx3#916) were seeded in 6-well plates for 48 h. BMDMs (4 × 10^6^ bone marrow cells incubated in 25 ng/ml M-CSF complete medium for 7 days of macrophage differentiation) were trypsinized and added to MEFs. After 48 h of co-culture, cells were harvested by using 5 mM EDTA and stained with fluorescence-conjugated antibodies including CD45, CD11b, F4/80, CD206, and CD86 for flow cytometric analysis.

### Bioinformatics analysis

For single-cell RNA-seq analysis, all data were analyzed and downloaded as png files on the Broad Institute Single Cell Portal (portals.broadinstitute.org/single_cell). The dataset named “Human Colon Cancer Atlas (c295)” was selected. For the scatter data and distribution data analysis, the settings were as follows: Gene name = PTX3, Clustering = All cells (tSNE), Annotation = ClusterTop, Subsampling = All Cells, Plot type = Violin plot, Data points = All.

The raw mRNA-seq data from the TCGA colon adenocarcinoma (COAD) dataset were obtained from the FireBrowse database (“http://firebrowse.org/”, [32]). In the mRNA-seq bar (Aliquot counts = 457), the illuminahiseq_rnaseqv2-RSEM_genes_normalized (MD5) and illuminaga_rnaseqv2-RSEM_isoforms_normalized (MD5) files were downloaded.

For gene set enrichment analysis (GSEA) (https://www.gsea-msigdb.org/gsea/index.jsp, [33]), the raw mRNA-seq data from the COAD data file [illuminahiseq_rnaseqv2-RSEM_genes_normalized (MD5)] were transformed into log2(raw counts + 1) values and split into the PTX3 high/low groups by the median cutoff value. The resulting file was uploaded into GSEA v.4.1.0 software for analysis. For KEGG pathway analysis, the c2.cp.kegg.v2023.1.Hs.symbols.gmt [Curated] gene set was selected. The signaling pathways enriched in the PTX3^high^ group are shown and were clustered into stromal activation-related signaling and inflammation-related signaling. The threshold criteria were NOM p value < 0.05 and FDR q value < 0.25. For inflammation response signature analysis, the HALLMARK_INFLAMMATORY_RESPONSE gene set was used.

For stromal score analysis, the stromal scores from the TCGA colorectal adenocarcinoma (RNA-seqV2) dataset were obtained from the ESTIMATE (Estimation of STromal and Immune cells in MAlignant Tumor tissues using Expression data) database (“https://bioinformatics.mdanderson.org/estimate/”, [34]) with the following parameters: Disease type = Colorectal Cancer, and Platform type = RNA-Seq-V2. The raw data were downloaded and merged with PTX3 mRNA expression (illuminaga_rnaseqv2-RSEM_isoforms_normalized (MD5)) data obtained from the FireBrowse database mentioned above. The correlation plot was generated by using GraphPad Prism 8.0 software.

For analysis of fibroblast scores, the preanalyzed TCGA colon adenocarcinoma data were downloaded via the xCell web tool (“https://xcell.ucsf.edu/”, [35]). The score data were merged with the PTX3 mRNA expression [illuminahiseq_rnaseqv2-RSEM_genes_normalized (MD5)] data obtained from the FireBrowse database mentioned above and split into the PTX3 high/low or fibroblast high/low groups by the median cutoff value.

For analysis of survival prognosis, survival data from the TCGA colon adenocarcinoma dataset was obtained via the SurvExpress web tool (“http://bioinformatica.mty.itesm.mx/SurvExpress”, [36], downloaded in 2018). The survival prognosis data were merged with the PTX3 mRNA expression and fibroblast score data mentioned above, and analysis results were plotted by using GraphPad Prism 8.0 software. The samples were split into high/low fibroblast score groups by the best cutoff value (114/89), and these groups were further subdivided into PTX3 high/low groups by the median cutoff value, and analysis results were plotted by using GraphPad Prism 8.0 software.

For analysis of the immune scores in the TCGA colon adenocarcinoma dataset, the raw mRNAseq data of COAD (illuminahiseq_rnaseqv2-RSEM_genes_normalized (MD5)) were split into the PTX3 high/low groups by the median cutoff value and uploaded to the CIBERSORT web tool (“https://cibersort.stanford.edu/”, [37]). The LM22 signature (22 immune cell types) was selected. The results of immune score analysis were plotted by using GraphPad Prism 8.0 software.

For analysis of RNA-seq data to predict PTX3 downstream signaling, the Connectivity Map web tool ("https://clue.io/about", [38]) was used. The top 200 upregulated and downregulated genes were uploaded in the “Query app” in the Tools section. The query parameters were as follows: Gene expression (L1000), Touchstone, Individual query, and 1.0. For the forward analysis, the upregulated genes were input in the UP-regulated genes box, and the downregulated genes were input in the DOWN-regulated genes box; after analysis, the Gene Over-Expression was selected as the perturbagen type, and candidates with a score of > 0 were obtained. For the reverse analysis, the upregulated genes were input in the DOWN-regulated genes box, and the downregulated genes were input in UP-regulated genes box; after analysis, the Gene Knock-Down was selected as the perturbagen type, and the candidates with a score of > 0 were obtained. We selected the overlapping candidates from the forward and reverse analysis results.

### Statistical analysis

Statistical analyses of all data in this study were performed by using GraphPad Prism 8.0, and data are presented as the means ± SEMs. Significant differences were identified by using two-tailed unpaired Student's t test, two-tailed paired Student's t test, one-way ANOVA or two-way ANOVA. GSEA data were analyzed by using GSEA_4.1.0. with the parameters set as p value of < 0.05 and FDR < 0.25. Statistical significance is represented as * p < 0.05, ** p < 0.01, *** p < 0.001. ns represents nonsignificant difference.

## Results

### PTX3 expression is associated with inflammation and stromal signatures in colon cancer

**Fig. 1:**
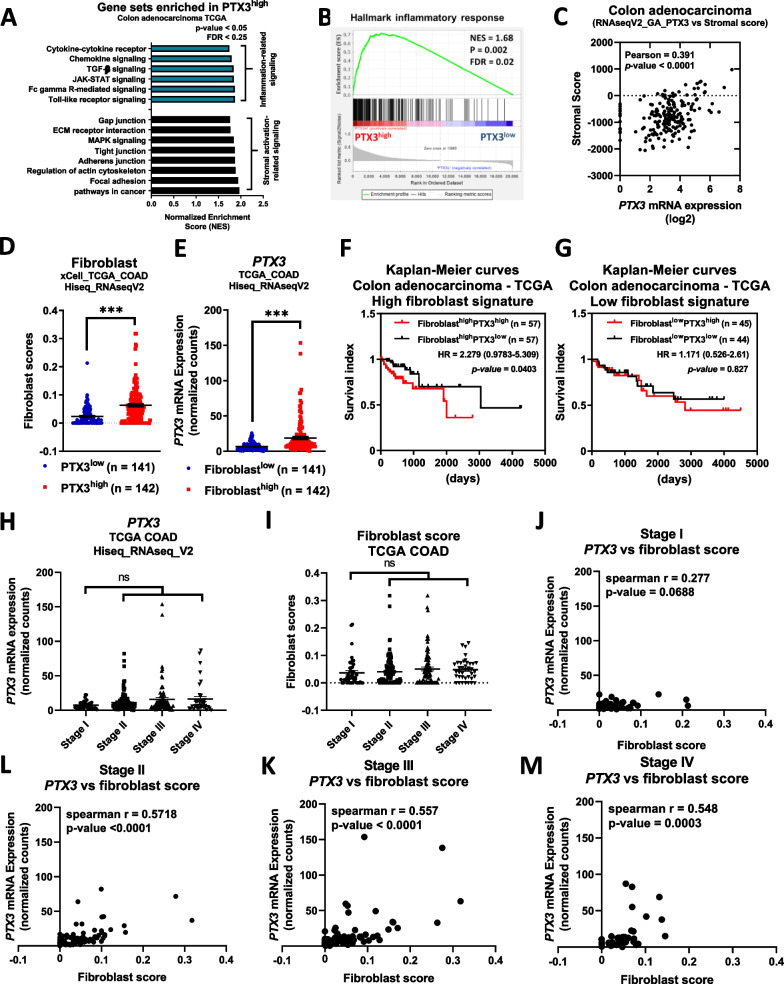
PTX3 expression is positively correlated with stromal and fibroblast signatures in colon cancer. A total of 287 colon adenocarcinoma samples represented in the TCGA dataset were divided into the PTX3^high^ (n = 143) and PTX3^low^ (n = 144) groups by the median cutoff value. **A** KEGG pathways related to cellular processes were selected for gene set enrichment analysis (GSEA), and the enriched signaling pathways (blue, inflammation-related signaling; and black, and stromal activation-related signaling) in PTX3^high^ tumors are shown. The threshold criteria were p value of < 0.05 and FDR < 0.25. **B** GSEA of inflammatory response signature in the PTX3^high^ (n = 143) and PTX3^low^ (n = 144) groups. The threshold criteria were NES > 1.5, p value of < 0.05, and FDR < 0.25. **C** The stromal scores of 191 colon adenocarcinoma samples from the TCGA dataset were extracted via the ESTIMATE web tool and used for Pearson correlation analysis with PTX3 expression levels (log2). **D**, **E** Fibroblast scores in 283 colorectal tumor samples from the TCGA dataset were extracted from the xCell database. The samples were divided into the PTX3^high^ (n = 142) and PTX3^low^ (n = 141) expression groups or the fibroblast^high^ (n = 142) and fibroblast^low^ (n = 141) score groups by the median cutoff value. **F**, **G** The survival index of 203 colorectal adenocarcinoma patients was obtained via the SurvExpress web-tool. The samples were divided into the fibroblast^high^ (n = 114) and fibroblast^low^ (n = 89) group by the best cutoff value, and each of these groups was subdivided into a PTX3^high^ group and a PTX3^low^ group by the median cutoff value. **H** PTX3 expression and **I** fibroblast scores in different stages in the TCGA COAD dataset are shown. Spearman correlation analysis of PTX3 expression and the fibroblast score in stage I (**J**), stage II (**K**), stage III (**L**), and stage IV (**M**) tumors. p values were calculated by two-tailed unpaired Student's t test, one-way ANOVA, two-sided log-rank test, HR hazard ratio, or CI confidence interval. ns represents nonsignificant difference. ***represents a p value of < 0.001. Values on the plots are presented as the means ± SEMs

The PTX3 level is increased in various chronic inflammatory diseases, and while elevated plasma PTX3 is linked to unfavorable prognosis in colorectal cancer [39], the primary secretory cells responsible for PTX3 production and the communication interactions of PTX3 within the tumor microenvironment remains unclear. To identify the primary cell types producing PTX3 in colon tumors, we analyzed single-cell data from the Human Colon Cancer Atlas (c295), which encompasses 371,223 malignant and nonmalignant cells, on the Single Cell Portal platform. These samples were categorized into seven clusters, with PTX3 expression predominantly enriched in myeloid cells and stromal cells (Additional file 3: Fig. S1A–C). Further subclustering of myeloid cells and stromal cells revealed that myeloid cells were classified into four subtypes, while stromal cells were classified into five subtypes. PTX3 was enriched primarily in monocytes, macrophages, and fibroblasts, with weak expression in granulocytes and endothelial cells (Additional file 3: Fig. S1D–I). This observation suggests an association between PTX3 expression and immune-related or stromal signatures. To validate the relationships between PTX3 and immune cells as well as stromal cells, we conducted gene set enrichment analysis (GSEA) and stromal score analysis in the TCGA COAD dataset. The results demonstrated enrichment of inflammation-related signaling pathways, stromal activation-related signaling pathways, and inflammatory responses in PTX3-high colon tumors compared to PTX3-low tumors (Fig. 1A, B). In the TCGA COAD dataset, PTX3 expression was also positively correlated with the stromal signature (Fig. 1C) as well as with the expression of proinflammatory genes, including interleukin-1β (IL1B) and interleukin-8 (IL8), and stromal extracellular matrix (ECM) marker genes, such as fibronectin 1 (FN1), collagen 1a2 (COL1A2), and actin alpha 2 (ACTA2) (Additional file 3: Fig. S2A).

To evaluate the impact of PTX3 expression and stromal scores on clinical outcomes in the TCGA COAD dataset, we performed an analysis with the xCell web tool to profile cell populations. PTX3-high colon tumors exhibited a high fibroblast signature score, and similarly, fibroblast-high colon tumors exhibited higher PTX3 expression **(**Fig. 1D and E), consistent with the results of single-cell data analysis that identified fibroblasts as the primary source cells of PTX3 in colon tumors. Combining these data with survival data, we found that the combination of a high fibroblast signature score with high PTX3 expression (fibroblast-high/PTX3-high signature) in tumors exhibited a more significant positive correlation with a poor survival prognosis than did the fibroblast-high/PTX3-low signature (Fig. 1F). Additionally, a positive correlation was observed between the fibroblast signature score and PTX3 expression in these samples (Additional file 3: Fig. S2B). In tumors with a low fibroblast signature score (fibroblast-low), PTX3 expression did not correlate with survival prognosis (Fig. 1G). Although neither PTX3 expression nor the fibroblast signature score was associated with an advanced stage of colon cancer in TCGA (Fig. 1H and I), PTX3 expression was positively correlated with the fibroblast signature score in stage 2, 3, and 4 tumors (Fig. 1J–M). These results suggest that PTX3 is predominantly expressed by fibroblasts and is linked to stroma-mediated late-stage progression in patients with colon cancer.

### PTX3 is silenced in colon tumor cells but highly expressed in stromal cells

**Fig. 2:**
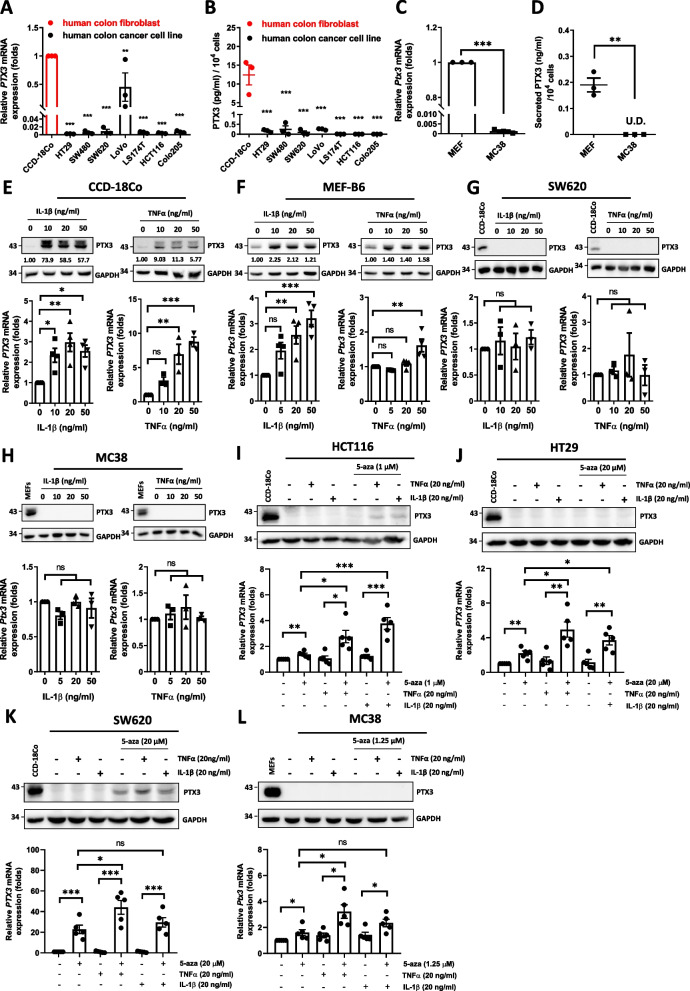
The expression of PTX3 is suppressed in colon cancer cells due to a regulation of epigenetic silencing. **A**, **B** The mRNA expression and secreted protein levels of PTX3 in CCD-18Co human colon fibroblasts, and 7 human colon cancer cell lines. **C**, **D** The mRNA expression and secreted protein levels of PTX3 in mouse embryonic fibroblasts (MEFs) and the mouse colon cancer cell line MC38. The conditioned medium was harvested for ELISA assay (n = 3). **E**–**H** PTX3 mRNA and protein in CCD-18Co cells, MEFs, SW620 cells, and MC38 cells were measured by real-time PCR and western blot, respectively. The experimental cells were treated with IL-1β or TNF-α for 48 h. **I**–**L** PTX3 mRNA and protein in HCT116, HT29, SW620, and MC38 cells were measured by real-time PCR and western blot, respectively. The experimental cells were treated with 5-azacytidine for 4 days and stimulated with 20 ng/ml IL-1β or TNF-α for 24 h beginning on the 3^rd^ day. GAPDH as internal control. p values were calculated by two-tailed unpaired Student’s t test or one-way ANOVA. ns represents nonsignificant difference. *represents a p value of < 0.05. **represents a p value of < 0.01. ***represents a p value of < 0.001. Values on the plots are presented as the means ± SEMs. Data are combined from 3 to 5 independent experiments

We subsequently conducted in vitro assessments to corroborate the bioinformatic analysis results, which indicated that PTX3 is primarily expressed by stromal cells. Notably, previous reports indicated that the PTX3 promoter tends to undergo extensive methylation and subsequent inactivation via epigenetic regulation in colon cancer cells [22, 40], shedding light on a possible explanation for our observations. We further investigated the differences in and inducibility of PTX3 expression in fibroblasts and colon cancer cell lines. Compared to those in CCD-18Co human colon fibroblasts and mouse embryonic fibroblasts (MEFs), the transcript and protein levels of PTX3 were very low or negligible in human and mouse colon cancer cell lines (Fig. 2A–D). PTX3 mRNA expression and protein levels were significantly induced by IL-1β and TNF-α in human and mouse fibroblasts (Fig. 2E and F) but not in colon cancer cells (Fig. 2G and H). Following treatment with 5-azacytidine, we found that the PTX3 transcripts were restored in cancer cells but IL-1β and TNF-α exerts a different effect on the induction of PTX3 gene in a cell type-specific manner. Notably, PTX3 protein levels were still too low to be detected in cancer cells under 5-azacytidine treatment, and only SW620 displayed detectable protein amounts (Fig. 2I–L). These results suggested the involvement of epigenetic regulation on the PTX3 gene in colon cancer cells and that stromal fibroblast is a major donor, at least, for PTX3 abundance.

### Inhibition of PTX3 reduces stroma-mediated progression of colon cancer

**Fig. 3:**
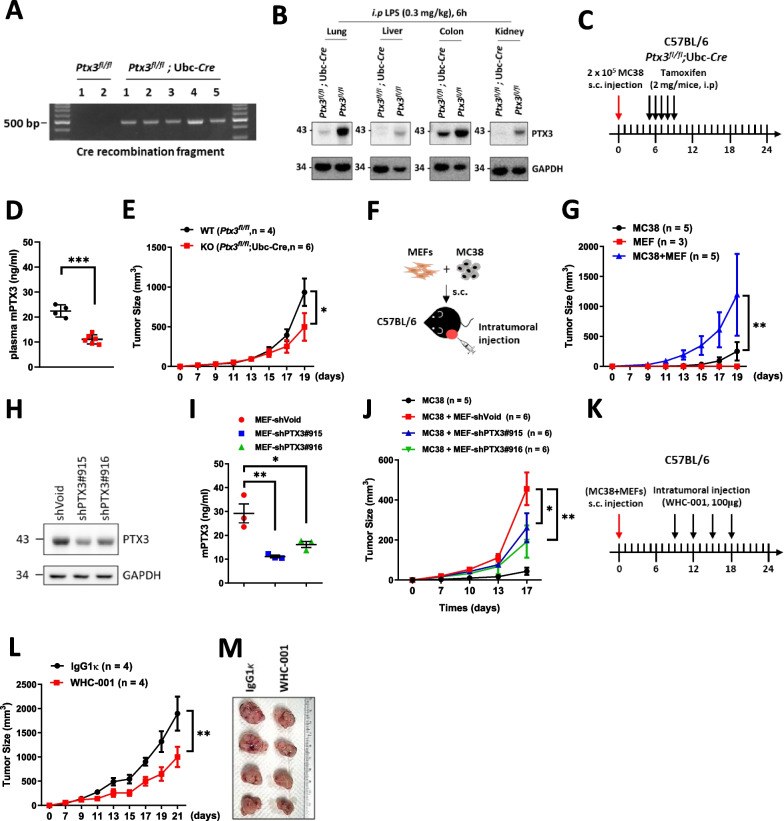
Inhibition of PTX3 reduces stroma-mediated tumor growth in vivo. Conditional knockout mice (*Ptx3*^*fl/fl*^ or *Ptx3*^*fl/fl*^; Ubc-Cre) were given tamoxifen (2 mg/mice, i.p) to drive Cre expression and **A** colon tissues were harvested for genotyping by detecting the recombined fragment (*Ptx3*^*fl/fl*^, n = 2 and *Ptx3*^*fl/fl*^; Ubc-Cre, n = 5). **B** Conditional knockout mice were given tamoxifen (2 mg/mouse, i.p) daily for 5 days, and after another 5 days, were treated with LPS (0.3 mg/kg, i.p) for 6 h. Lung, liver, colon, and kidney tissues were harvested to measure the Ptx3 protein levels (*Ptx3*^*fl/fl*^, n = 1 and *Ptx3*^*fl/fl*^; Ubc-Cre, n = 1). **C** The experimental design for the in vivo tumor growth of MC38 by latter knockout of *Ptx3* in conditional knockout mice (*Ptx3*^*fl/fl*^, n = 4 and *Ptx3*^*fl/fl*^; Ubc-Cre, n = 6). Tamoxifen was given 5 days after MC38 cell inoculation, and **D** the plasma Ptx3 level was measured and **E** tumor growth curves were generated. **F**, **G** Illustration and growth curves of subcutaneous tumors formed from 5 × 10^4^ MC38 cells (n = 5), 1 × 10^5^ MEFs (n = 3), or 5 × 10^4^ MC38 cells mixed with 1 × 10^5^ MEFs (n = 5) in C57BL/6 mice. **H**, **I** Intracellular and secretory Ptx3 protein levels in shVoid, sh*Ptx3*#915, and sh*Ptx3*#916 MEFs. Conditioned medium was harvested for ELISA assay (n = 3). **J** The growth curves of subcutaneous tumors formed from MC38 cells (n = 5) and MC38 cells mixed with MEFs-shVoid (n = 6), -sh*Ptx3*#915 (n = 6), or -sh*Ptx3*#916 (n = 6). **K–M** Illustration and growth curves of subcutaneous tumors in the MC38 cell/MEF tumor model. Intratumoral administration of IgG1κ (n = 4) or WHC-001 (αPTX3 Ab, n = 4) (100 μg) was performed every 3 days for a total of 4 treatments beginning when the tumor volume reached to 80 mm^3^. Tumor volume (mm^3^) = (L × W^2^)/2. p values were calculated by two-tailed unpaired Student’s t test or one-way ANOVA. *represents a p value of < 0.05. **represents a p value of < 0.01. ***represents a p value of < 0.01. Values on the plots are presented as the means ± SEMs

Although PTX3 expression is attenuated in colon cancer cells, it has been shown that increased plasma PTX3 is positively correlated with a poor survival outcome [41]. Moreover, based on our observation that the positive association between PTX3 expression and stromal cells is linked to unfavorable survival outcomes in colorectal cancer patients, we proposed that PTX3 plays a role in stroma-mediated tumor progression. To investigate the involvement of PTX3 in and its effects on stroma-mediated colon tumor growth, we employed tamoxifen-induced *Ptx3* conditional knockout mice [*Ptx3*^*fl/fl*^ and *Ptx3*^*fl/fl*^;Ubc-Cre mice, previously established by our group [29]]. Following tamoxifen administration, the *Ptx3* gene was systemically deleted, as confirmed by measuring the expression of the recombined fragment of *Ptx3* genomic DNA (Fig. 3A) and the Ptx3 protein level in various organs (Fig. 3B). To assess the effects of stromal PTX3 in an allograft model, we utilized MC38 cells in *Ptx3* systemic knockout mice, as MC38 cells exhibit very low levels of *Ptx3* expression. Subcutaneous injection of MC38 cells into *Ptx3*^*fl/fl*^ or *Ptx3*^*fl/fl*^;Ubc-Cre mice was followed by tamoxifen administration 5 days later (Fig. 3C). The knockout efficiency was examined by measuring the plasma Ptx3 level in both *Ptx3*^*fl/fl*^ and *Ptx3*^*fl/fl*^;Ubc-Cre mice (Fig. 3D), and the results showed that under systemic *Ptx3* deletion, MC38 tumor growth was attenuated (Fig. 3E), suggesting that PTX3 contributes to tumor progression**.**

In addition to using tamoxifen-induced *Ptx3* conditional knockout mice, we established a subcutaneous mixed stroma–tumor model in mice by coinjecting MEFs (high PTX3-expressing stromal cells) and MC38 cells (low PTX3-expressing cancer cells) (Fig. 3F). Coinjection of MEFs significantly increased tumor growth in vivo compared with that in the group injected with only MC38 cells (Fig. 3G). To assess whether PTX3 is involved in MEF-mediated tumor growth, we coinjected *Ptx3* knockdown MEFs (sh*Ptx3*#915, sh*Ptx3*#916) or control MEFs (shVoid) and monitored MC38 tumor growth in vivo. The *Ptx3* knockdown efficiency in MEFs was validated by measuring the intracellular and secreted protein levels (Fig. 3H and I). Our results demonstrated that coinjection of *Ptx3* knockdown MEFs led to a reduction in MC38/MEF tumor growth (Fig. 3J), suggesting a contribution of PTX3 to stroma-mediated tumor growth. Furthermore, we established a therapeutic model by administering an anti-PTX3 antibody [WHC-001; a neutralizing antibody that binds to PTX3, preventing its interaction with CD44 [29]] to evaluate whether blocking PTX3 in the tumor microenvironment can mitigate stroma-mediated tumor growth. In the MC38/MEF mouse model, intratumoral administration of WHC-001 attenuated MC38/MEF allograft tumor growth in vivo (Fig. 3K–M).

**Fig. 4:**
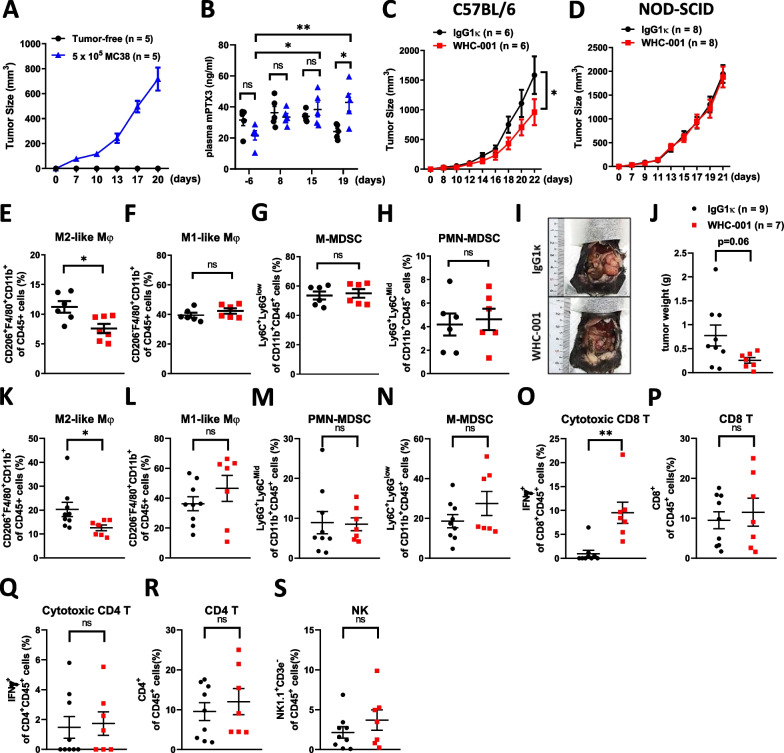
Blockade of PTX3 reduces tumor growth and the population of tumor-infiltrating M2-like macrophages and increases the population of tumor-infiltrating cytotoxic CD8^ + ^T cells. **A** The tumor growth curves and **B** plasma Ptx3 levels in MC38 tumor-bearing mice (n = 5) and tumor-free mice (n = 5). **C** The growth curves of subcutaneous MC38 tumors in immunocompetent mice (C57BL/6) following i.p. administration of IgG1κ (n = 6) or WHC-001 (αPTX3 Ab, n = 6) (10 mg/kg) every 4 days for a total of 3 treatments. **D** The growth curves of subcutaneous MC38 tumors in immunodeficient mice (NOD-SCID) following i.p. administration of IgG1κ (n = 8) or WHC-001 (αPTX3 Ab, n = 8) (10 mg/kg) every 4 days for a total of 3 treatments. Tumor volume (mm^3^) = (L × W^2^)/2. **E–H** Tumor-infiltrating immune populations of MC38 tumors in Fig. 4C were analyzed by flow cytometry. The CD45 positive immune cells were gated on PMN-MDSCs (Ly6G^ + ^Ly6C^Mid^/CD11b^+^), M-MDSCs (Ly6C^ + ^Ly6C^low^/CD11b^ +^), M1-like macrophages (CD11b^ + ^F4/80^ + ^CD206^−^), and M2-like macrophages (CD11b^ + ^F4/80^ + ^CD206^ +^). **I** The orthotopic colon tumors formed from 1 × 10^6^ MC38 cells in the cecal wall following i.p. administration of IgG1κ (n = 9) or WHC-001 (αPTX3 Ab, n = 7) (10 mg/kg) beginning on the 7^th^ day post tumor cell inoculation and repeated every 4 days for a total of 3 treatments. **J **The tumor weights and **K**–**S** tumor-infiltrating immune populations of MC38 tumors in Fig. 4J are shown. The CD45 positive immune cells were gated on CD8^ + ^T cells (CD8^ +^), CD4^ + ^T cells (CD4^ +^), cytotoxic CD8^ + ^T cells (IFNγ^ + ^CD8^ +^), NK cells (NK 1.1^ + ^CD3e^−^), PMN-MDSCs (Ly6G^ + ^Ly6C^Mid^/CD11b^ +^), M-MDSCs (Ly6C^ + ^Ly6C^low^/CD11b^ +^), M1-like macrophages (CD11b^ + ^F4/80^ + ^CD206^−^), and M2-like macrophages (CD11b^ + ^F4/80^ + ^CD206^ +^). p values were calculated by two-tailed unpaired Student’s t test, one-way ANOVA, or two-way ANOVA. *represents a p value of < 0.05. ns represents nonsignificant differences, *represents a p value of < 0.05, and **represents a p value of < 0.01. Values on the plots are presented as the means ± SEMs

Although MC38 cells exhibit a very low level of Ptx3 expression, mice bearing MC38 tumors exhibited higher levels of plasma Ptx3 than mice without a tumor burden (Fig. 4A and B). This suggests that the formation of MC38 tumors triggers an inflammatory-like response, resulting in increased Ptx3 production by stromal cells. Subsequently, we evaluated whether the blockade of Ptx3 with WHC-001 can also attenuate MC38 tumor growth when MEFs were not coinjected. Surprisingly, the results indicated that treatment with WHC-001 still inhibited MC38 tumor growth (Fig. 4C), suggesting that PTX3 is a potential therapeutic target in colon cancer.

To gain insight into how PTX3 contributes to stroma-mediated tumor growth, we initially explored whether PTX3 can directly promote the proliferation of cancer cells. However, we discovered that the administration of recombinant PTX3 protein had no discernible effect on the proliferation of either cancer cells or fibroblasts. Furthermore, colony formation by cancer cells remained unaffected, and we observed very low cytotoxicity of WHC-001 in both cancer cells and fibroblasts (Additional file 3: Fig. S3A–C). These findings suggest that inhibition of PTX3 attenuates tumor growth in vivo by disrupting a protumor effect in the tumor microenvironment.

### PTX3 contributes to tumor growth by regulating tumor immunity

Because PTX3 exerted no effect on cell proliferation, we then investigated whether PTX3 affects tumor growth by modulating the protumor microenvironment. Based on several reports demonstrating the role of PTX3 in regulating inflammation [42], we speculated that PTX3 contributes to tumor progression by mediating tumor immunity. To assess this hypothesis, we examined whether blockade of PTX3 still affects MC38 tumor growth in immunodeficient mice. The results showed that the administration of WHC-001 had no significant effect on MC38 tumor growth in immunodeficient mice (Fig. 4D). This observation implies that immunity may play a crucial role in PTX3-mediated tumor growth.

Tumor-infiltrating myeloid cells within the tumor microenvironment have been suggested to play a role in modulating tumor growth [43]. These myeloid cells include polymorphonuclear myeloid-derived suppressor cells (PMN-MDSCs), mononuclear myeloid-derived suppressor cells (M-MDSCs), and M1 and M2 macrophages. Given that PTX3 has been reported to enhance polarization toward the anti-inflammatory macrophage phenotype [30, 31], we hypothesized that PTX3 might regulate the phenotypes of myeloid cells in colon tumors. To investigate this possibility, we conducted an analysis of tumor-infiltrating myeloid populations in MC38 tumors using flow cytometry. The results revealed that treatment with WHC-001 reduced the number of tumor-infiltrating M2-like macrophages, but it had no significant impact on other myeloid populations (Fig. 4E–H; Additional file 3: Fig. S4A). In an orthotopic colon tumor model, blockade of PTX3 resulted in inhibited tumor growth (Fig. 4I and J), and this effect was also associated with a reduction in the number of tumor-infiltrating M2-like macrophages, while other myeloid populations, such as M1-like macrophages, PMN-MDSCs, and M-MDSCs, remained unaffected (Fig. 4K–N; Additional file 3: Fig. S4B). M2 macrophages have immunosuppressive effects that inhibit the cytotoxic activity of CD8^+^ T cells [44]. Our data indicated that WHC-001 increased the number of cytotoxic CD8^+^ T cells within tumors, although it had no significant effect on the total number of CD8^+^ T cells (Fig. 4O and P). This suggests that PTX3 contributes to the immunosuppressive conditions in the microenvironment of colon cancer. Furthermore, we analyzed other lymphocyte populations, including cytotoxic CD4^+^ T cells, total CD4^+^ T cells, and NK cells and found that they were not affected by WHC-001 treatment in tumors (Fig. 4Q–S). In addition to MC38 colon cancer cells of C57BL/6 origin, we employed CT26 colon carcinoma cells of BALB/c origin to validate the anticancer efficacy of WHC-001. The results showed that WHC-001 also exerted a suppressive effect on the growth of subcutaneous CT26 tumors (Additional file 3: Fig. S5A–C), which was associated with a decrease in the tumor-infiltrating M2-like macrophage population and an increase in the cytotoxic CD8^+^ T-cell population. Similarly, the populations of other myeloid cells and lymphocytes were not affected (Additional file 3: Fig. S5D–L). In our analysis of the colon cancer TCGA dataset, we observed that PTX3-high tumors expressed higher levels of the M2 macrophage signature genes and lower levels of the CD8^+^ T-cell signature genes (Additional file 3: Fig. S6), implying that PTX3 contributes to a protumor immune microenvironment.

### PTX3 promotes M2-like macrophage polarization

**Fig. 5:**
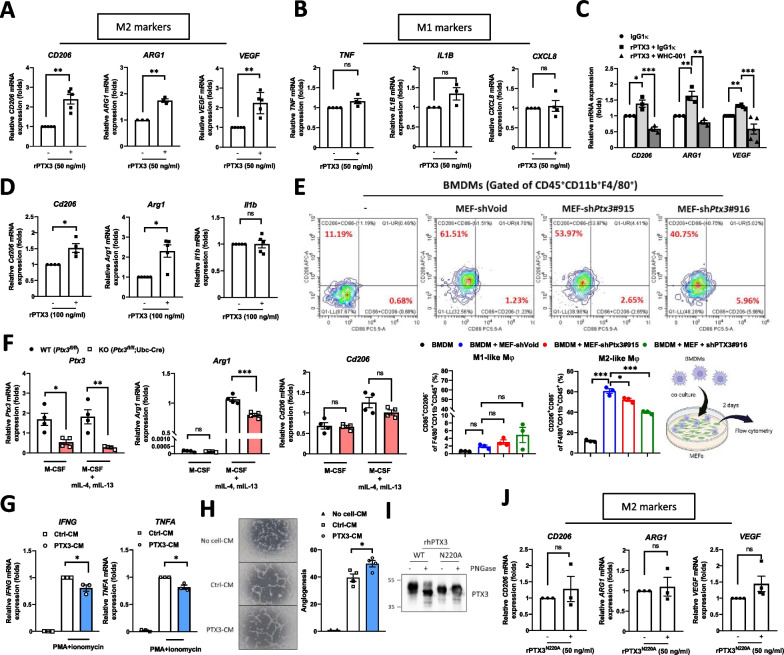
PTX3 promotes M2-like macrophage polarization. **A**, **B** The mRNA expression of M2 and M1 markers in THP-1 macrophages treated with or without 50 ng/ml PTX3. **C** The mRNA expression of M2 markers in PTX3-treated THP-1 macrophages with 250 ng/ml IgG1κ or WHC-001 (αPTX3Ab). **D** The mRNA expression of *Arg1*, *Cd206*, and *Il1b* in bone marrow-derived macrophages treated with or without 100 ng/ml PTX3. **E** Flow cytometric analysis of M1- and M2-like macrophages in BMDMs co-cultured with MEFs-shVoid, -sh*Ptx3*#915,or sh*Ptx3*#916 for 48 h. The CD45^ + ^CD11b^ + ^F4/80^ + ^ cells were characterized by M1-like (CD86^ + ^CD206^−^) and M2-like (CD206^ + ^CD86^−^) macrophages through flow cytometric analysis. **F** The mRNA expression of *Ptx3*, *Arg1*, and *Cd206* in tamoxifen-induced *Ptx3*^*fl/fl*^ or *Ptx3*^*fl/fl*^; Ubc-Cre bone marrow-derived macrophages (M-CSF-primed M0 BMDMs) following stimulation with 20 ng/ml mIL-13 and 20 ng/ml mIL-4 (M2 polarization). **G** The mRNA expression of *IFNG* and *TNFA* in activated Jurkat cells (stimulated with PMA + ionomycin) incubated with conditioned medium from untreated THP-1 macrophages (Ctrl-CM) or PTX3-treated THP-1 macrophages (PTX3-CM). **H** Angiogenesis of HUVECs incubated in serum-free medium (No cell-CM), conditioned-medium from untreated THP-1 macrophages (Ctrl-CM) or conditioned medium from PTX3-treated THP-1 macrophages (PTX3-CM). The relative angiogenic ability was determined by the number of branch sites/nodes of tubes per field of view. **I** Western blot analysis of recombinant wild-type and N220A mutant PTX3 proteins treated with or without PNGase. **J** The mRNA expression of M2 markers in THP-1 macrophages treated with or without 50 ng/ml recombinant N220A mutant PTX3 protein. p values were calculated by two-tailed paired Student’s t test, one-way ANOVA, or two-way ANOVA. ns represents nonsignificant difference,*represents a p value of < 0.05, **represents a p value of < 0.01, and ***represents a p value of < 0.001. Values on the plots are presented as the means ± SEMs. Data are combined from 3 to 5 independent experiments

We next investigated whether PTX3 directly promotes the differentiation of macrophages toward an M2-like phenotype. In THP-1 macrophages (PMA-primed THP-1 cell-derived M0 macrophages), PTX3 induced the expression of M2 markers (*ARG1*, *CD206*, and *VEGF*) (Fig. 5A) but had no effect on M1 markers (*IL1B*, *CXCL8*, and *TNFA*) **(**Fig. 5B**)**. WHC-001 is a neutralizing antibody developed to disrupt the binding of PTX3 to CD44, a key receptor that also contributes to M2 polarization of THP-1 cells [45]. Using an in situ proximity ligation assay (PLA), we showed that WHC-001 attenuated the binding of PTX3 to CD44 on THP-1 macrophages (Additional file 3: Fig. S7A–C). We then assessed the capability of WHC-001 to inhibit PTX3-induced expression of M2 markers in THP-1 macrophages. The results showed that treatment with WHC-001 attenuated the increase in M2 marker expression in PTX3-treated THP-1 macrophages (Fig. 5C), suggesting the involvement of a PTX3/CD44 axis in M2 polarization. Subsequently, we explored the capability of PTX3 to drive the expression of M2 markers in primary mouse bone marrow-derived macrophages (BMDMs). Similarly, administration of recombinant murine Ptx3 protein upregulated *Arg1* and *Cd206* expression (M2 markers) but did not affect *Il1b* expression (M1 markers) (Fig. 5D). To verify the effect of stromal cells-derived PTX3 on induction of M2 macrophage polarization, BMDMs were co-cultured with control (shVoid) or *Ptx3* knockdown (sh*Ptx3*#915, sh*Ptx3*#916) MEFs to analyze M1- and M2-like macrophage polarization by flow cytometry. The results showed that co-culture of BMDMs with MEFs significantly elevated M2 macrophages polarization, and this effect was attenuated in the group of BMDMs co-cultured with *Ptx3* knockdown MEFs (Fig. 5E), indicating the vital role of stromal PTX3 to promote M2 macrophage polarization. Next, BMDMs isolated from *Ptx3*-knockout mice were utilized to verify the effect of PTX3 autoregulation on M2 macrophage polarization. Compared to that in M0 macrophages, *Ptx3* expression was unaffected in M2-polarized macrophages (stimulated by IL-4 and IL-13). We further found that *Arg1* expression, but not *Cd206* expression, was reduced in *Ptx3-*knockout M2-polarized BMDMs (Fig. 5F), suggesting that PTX3 has a minor autoregulatory effect on M2 polarization of macrophages.

M2 macrophages are known to suppress the cytotoxic activity of CD8^+^ T cells, leading to immunosuppression within the tumor microenvironment. Indeed, we observed that treatment with conditioned medium derived from PTX3-treated THP-1 macrophages reduced the expression of interferon gamma (*IFNG*) and tumor necrosis factor alpha (*TNFA*) in activated Jurkat cells (Fig. 5G), suggesting that PTX3-treated macrophages can inhibit the cytotoxic activity of T cells. However, PTX3 had no direct effect on the regulation of *IFNG* and *TNFA* transcription in Jurkat cells (Additional file 3: Fig. S8A–D). Furthermore, we examined other aspects of the M2 phenotype in PTX3-treated THP-1 macrophages, such as angiogenesis. The results showed that treatment with conditioned medium from PTX3-treated THP-1 macrophages increased the tube formation ability of HUVECs (Fig. 5H). These findings consistently support the hypothesis that PTX3 promotes M2 macrophage polarization and potentially contributes to the establishment of a protumor environment. In addition, because N-glycosylation at Asn220 is important for the function of PTX3 [42] (Fig. 5I), we sought to investigate whether this modification affects PTX3-mediated M2 macrophage polarization. In comparison to the response elicited by wild-type PTX3, the induction of M2 markers was attenuated in THP-1 macrophages treated with N220A PTX3 (Fig. 5J). This result suggests that N-glycosylation of PTX3 plays a vital role in regulating M2 macrophage polarization.

### The CREB1/CEBPB axis responds to PTX3 and mediates M2 macrophage polarization

**Fig. 6:**
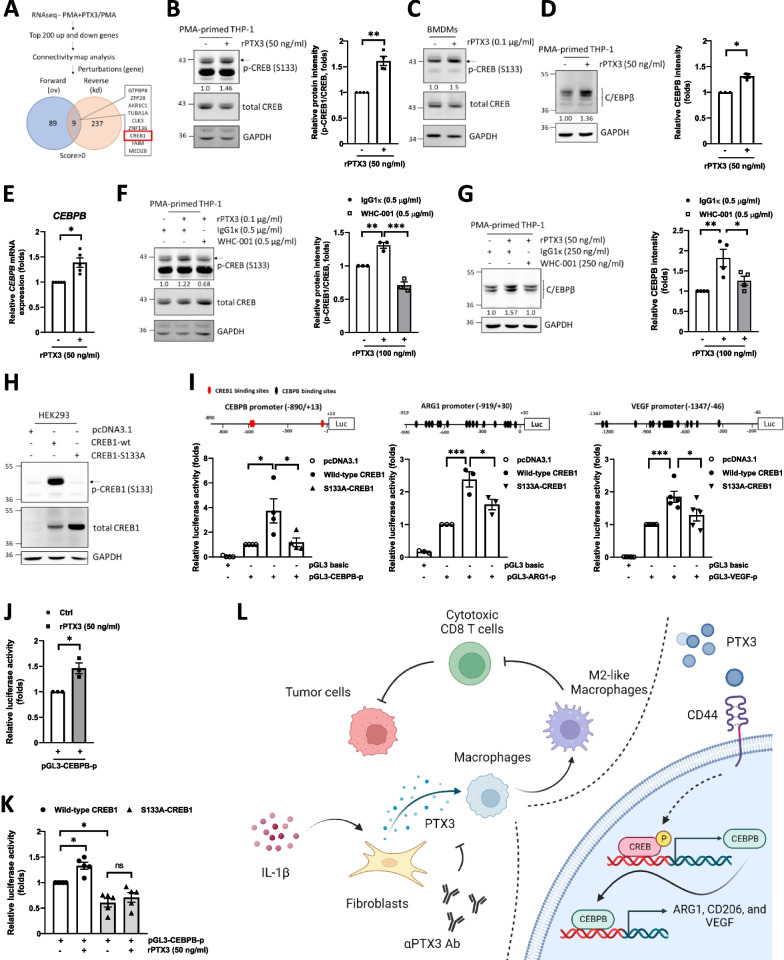
PTX3 contributes to M2 polarization through activation of CREB1 signaling. **A** RNA sequencing was performed in unstimulated THP-1 macrophages and PTX3-stimulated THP-1 macrophages. The top 200 upregulated and downregulated genes were selected for Connectivity Map analysis. Forward (overexpression) and reverse (knockdown) perturbation candidate genes were identified. **B** Western blot analysis of p-CREB1 and CREB1 in THP-1 macrophages treated with or without 50 ng/ml PTX3. **C** Western blot analysis of p-CREB1 and CREB1 in BMDM streated with or without 100 ng/ml PTX3. **D** The protein level and **E** mRNA level of CEBPB in THP-1 macrophages treated with or without 50 ng/ml PTX3. **F**, **G** The protein levels of p-CREB1, CREB1, and CEBPB in THP-1 macrophages treated with 100 ng/ml PTX3 plus either 500 ng/ml IgG1κ or WHC-001 (αPTX3Ab). **H** Western blot analysis of p-CREB1 and CREB1 in HEK293 cells transiently transfected with pcDNA3.1, CREB1-wt, or CREB1-S133A. **I** Illustration of the luciferase promoter constructs of CEBPB (− 890/ + 13), VEGF (− 1347/− 46), and ARG1 (− 919/ + 30). The CREB1 binding site is indicated in red and the CEBPB binding site is indicated in black. The promoter activity of the pGL3-CEBPB (− 890/ + 13), pGL3-VEGF (− 1347/− 46), and pGL3-ARG1 (− 919/ + 30) constructs in HEK293 cells cotransfected with pcDNA3.1, CREB1-wt, or CREB1-S133A is shown. **J** The promoter activity of pGL3-CEBPB (− 890/ + 13) in THP-1 macrophages treated with or without 50 ng/ml PTX3. **K** The promoter activity of pGL3-CEBPB (− 890/ + 13) in THP-1 macrophages cotransfected with CREB1-wt or CREB1-S133A following treatment with or without 50 ng/ml PTX3. **L** Schematic model showing that PTX3 contributes to stroma-mediated immunosuppression by promoting M2-like macrophage polarization through activation of the CEBPB/CREB1 axis in colon cancer. p values were calculated by two-tailed paired Student’s t test or one-way ANOVA. ns represents nonsignificant difference, *represents a p value of < 0.05, **represents a p value of < 0.01, and ***represents a p value of < 0.001. Values on the plots are presented as the means ± SEMs. Data are combined from 3 to 5 independent experiments

To elucidate the downstream effectors activated in response to PTX3, we conducted RNA-seq analysis using transcripts from PTX3-treated and untreated THP-1 macrophages. The top 200 upregulated and top 200 downregulated individual genes were subjected to Connectivity Map analysis [38] to predict perturbation genes (potential genes that lead to gene candidate expression patterns similar to the input patterns). This analysis identified nine candidates, including CREB1 and ZNF136, as common upstream regulators activated in response to PTX3-regulated upregulated and downregulated genes (Fig. 6A). Given that the CREB1/CEBPB axis has been previously implicated in M2 macrophage polarization, we further examined whether the CREB1/CEBPB axis responds to PTX3 treatment and mediates PTX3-induced M2 macrophage polarization. We found that treatment with PTX3 increased the activation of CREB1 (Fig. 6B and C) and upregulated the expression of CEBPB (Fig. 6D and E) in macrophages. In loss-of-function assays, WHC-001 attenuated PTX3-induced CREB1 activation (Fig. 6F) and CEBPB expression (Fig. 6G). Following the examination of CREB1 and mutant CREB1^S133A^ activation in HEK293 cells (Fig. 6H), we examined whether CREB1 activity is crucial for PTX3-mediated M2 marker transcription. Compared to that in CREB1 transfectants, the activity of the *CEBPB*, *VEGF,* and *ARG1* reporters was attenuated in mutant CREB1^S33A^ transfectants (Fig. 6I). Furthermore, we conducted tests to determine whether CREB1 activity mediates PTX3-induced CEBPB transcription in THP-1 macrophages. The results showed that the activity of the *CEBPB* reporter was responsive to PTX3 treatment in THP-1 macrophages (Fig. 6J) and that this induction was attenuated in CREB1^S133A^ transfectants (Fig. 6K). These results suggested that CREB1 activation is responsive to PTX3 and consequently contributes to the induction of *CEBPB*, *ARG1* and *VEGF* transcription.

In summary, our findings reveal that (1) PTX3 expression tends to be silenced in colon tumor cells following tumorigenesis but can be sustained at high levels in stromal cells, (2) blockade of PTX3 with the specific antibody WHC-001 attenuates tumor growth by increasing the population of tumor-infiltrating cytotoxic CD8^+^ T cells and decreasing the population of tumor-infiltrating M2-like macrophages, and (3) the CREB1/CEBPB axis mediates the PTX3-induced promotion of M2 macrophage polarization (Fig. 6L).

## Discussion

Several studies have demonstrated that PTX3 expression is associated with progression in various types of cancers, such as head and neck cancer, breast cancer, gastric cancer, melanoma, hepatocellular carcinoma, and cervical cancer. Our recent work revealed that in breast cancer, a high level of PTX3 in CAFs was associated with a poor prognosis for survival and that PTX3 contributed to drug resistance, stemness, and metastasis [29]. In colon cancer, except for the observations of the epigenetic regulation of PTX3 gene expression (22) and the association of a higher plasma PTX3 level with a poor survival prognosis [39], the precise clinical and cellular effects of PTX3 expression remain unclear. In this study, we first demonstrated that PTX3 is mainly expressed by stromal cells and contributes to immunosuppression through activation of M2 macrophages in colon cancer.

Throughout tumorigenesis, numerous genes have been implicated in playing both protumor and antitumor roles. For instance, TGFβ, initially considered an anti-inflammatory cytokine, switches from functioning as a tumor suppressor in early stages to a tumor promoter in later stages of cancer progression [46]. Early knockout of the *TGFβ* gene promotes tumor growth [47, 48], and later knockout of the *TGFβ* gene inhibits tumor growth [49]. Nitric oxide (NO), a small molecule synthesized by nitric oxide synthase (iNOS), regulates various biological functions and exerts antitumor or protumor effects depending on its abundance [50, 51]. Interleukin-17 (IL-17) promotes angiogenesis to benefit tumor metastasis and potentiates the generation of cytotoxic effectors to kill cancer cells [52]. PTX3 has been reported to exert complex effects on cancer progression. In support of its antitumor role, conventional *Ptx3*-deficient mice exhibit an increased incidence of carcinogen-induced tumorigenesis [22], and PTX3 contributes to antitumor functions by suppressing EMT and angiogenesis [53, 54]. However, more studies have shown that PTX3 plays a protumor role by promoting drug resistance, metastasis, and stemness [23, 25–29, 55]. In this study, we offered a new perspective on how PTX3 modulates immunosuppression by promoting M2 macrophage polarization in the context of colon cancer.

We hypothesized that in addition to playing the abovementioned dual roles, PTX3 potentially exerts antitumor effects in the acute inflammation-like state (the early stage of tumorigenesis) but acts as a tumor promoter in the chronic inflammation-like state (the late stage of tumorigenesis). PTX3 has been reported to induce pyroptosis [56]. In the antitumor role of pyroptosis, pyroptosis-induced inflammasome activation suppresses tumorigenesis in colitis-associated colon cancer [57–59]. Conversely, in its protumor role, pyroptosis can lead to the chronic accumulation of proinflammatory factors, fostering cancer cell proliferation, angiogenesis, metastasis, and immunosuppression [60]. Hence, it is imperative to investigate whether PTX3-induced pyroptosis plays a functional role in tumorigenesis, particularly in the context of its association with PTX3-induced anti- and protumor effects during the progression of colon cancer. Further research is needed to elucidate the mechanistic details of this phenomenon and its relevance to cancer development.

PTX3 regulates diverse cellular processes, but one critical question remains: Which membrane receptor is critical for PTX3 binding and mediates the protumor role of PTX3? In previous studies, PTX3 was shown to directly bind to FcγR and induce the phagocytosis of pathogens by neutrophils [61, 62]. Dectin-1 and Toll-like receptor 4 have also been suggested as potential PTX3-binding membrane receptors [26, 63]. We recently identified CD44, a nonkinase transmembrane glycoprotein, as a receptor that directly binds PTX3 and mediates PTX3-induced protumor effects in triple-negative breast cancer [29]. CD44 is a common coreceptor that participates in several signaling pathways and is involved in tumor progression [64]. The results of our experiments with the PTX3 inhibitor WHC-001 supported the idea that disruption of the PTX3/CD44 interaction suppresses colon cancer progression in vivo. However, several unresolved issues, including (1) whether other potential PTX3 receptor contribute to PTX3-mediated protumor effects, (2) whether WHC-001 disrupts other potential PTX3 receptor interactions and (3) whether CD44 is the critical factor in the switch between the anti- and protumor role of PTX3 in colon cancer progression, remain to be further investigated.

In this study, we demonstrated that the CREB1/CEBPB axis, at least in part, mediates the PTX3-induced promotion of M2 macrophage polarization. Moreover, WHC-001 can inhibit PTX3-induced CREB1 S133 phosphorylation. A previous study showed that the intracellular domain of CD44 interacts with CREB1 to increase CREB1 phosphorylation [65], suggesting that CD44 may mediate PTX3-induced activation of the CREB1/CEBPB axis in macrophages. Signaling in response to CD44 activation has been suggested to promote M2 macrophage polarization [66–68]. Therefore, exploring the crosstalk among and independence of these CD44-interacting proteins involved in M2 macrophage polarization and their contributions to colon tumorigenesis could be a valuable avenue for future research. Additionally, CD44 has been linked to the regulation of regulatory T cells (Tregs), which also play a role in immunosuppression by producing the anti-inflammatory cytokine TGFβ, thereby suppressing CD8^+^ T-cell cytotoxicity [69]. Moreover, CD44 costimulation has been shown to favor Treg functions, and CD44-knockout mice exhibit impaired Treg activity [70]. Taken together, in addition to the discovery of the direct effect of PTX3 on cancer cells [29] and macrophages, recent evidence potentially supports an effect of CD44 and PTX3 on regulating Treg activation or differentiation.

## Conclusions

In conclusion, our study revealed that increased or sustained PTX3 expression in the stroma, particularly in fibroblasts, contributes to colon tumorigenesis. Our investigation into PTX3's role in promoting M2 macrophage polarization in colon cancer sheds light on its functional involvement in modulating immunosuppression within the tumor microenvironment. Furthermore, beyond the previously identified potential of the PTX3 inhibitor WHC-001 for suppressing metastasis, invasion, stemness, and drug resistance in triple-negative breast cancer [29], our findings reveal a novel effect of WHC-001 on reversing immunosuppression by reducing the population of tumor-infiltrating M2 macrophages and increasing the population of tumor-infiltrating cytotoxic CD8^+^ T cells in colon tumors. Therefore, targeting PTX3 with WHC-001 emerges as a promising therapeutic strategy for colon cancer.

### Supplementary Information

**Additional file 1: Table S1.** The primers for real-time polymerase chain reaction (real-time PCR).

**Additional file 2: Table S2.** The primers for plasmid construction.

**Additional file 3: Figures. S1**–**S8**.

**Additional file 4: **Additional materials and methods.

## Data Availability

All data generated in this study are available in the main text and additional information files. Additional Tables are attached in Additional files 1 and 2. The Supplementary figures are shown in Additional file 3: Figs. S1–S8. The additional materials and methods are attached in Additional file 4. The RNAseq data in this study are available in Gene Expression Omnibus (GEO) database [71] at GSE227402. The availability of data from open-access databases is described in “Bioinformatics analysis” section of Methods. Due to the SurvExpress web-tool has been closed, the raw data of survival analysis in this study are available upon reasonable request from the corresponding author.

## Abbreviations

PTX3: Pentraxin 3
N220A: Asparagine at 220 to Alanine
CD44: CD44 molecule
CAF: Cancer-associated fibroblast
CREB1: Cyclic AMP-responsive element-binding protein 1
S133A: Serine at 133 to Alanine
CEBPB: CCAAT/enhancer-binding protein beta
MDSCs: Myeloid-derived suppressor cells
TANs: Tumor-associated neutrophils
TAMs: Tumor-associated macrophages
TNFA: Tumor necrosis factor alpha
IL1B: Interleukin 1 beta
IFNG: Interferon gamma
ARG1: Arginase 1
VEGF: Vascular endothelial growth factor
FN1: Fibronectin 1
COL1A2: Collagen type I alpha 2 chain
ACTA2: Actin alpha 2, smooth muscle
ZNF136: Zinc finger protein 136
TCGA: The cancer genome atlas
COAD: Colon adenocarcinoma
KEGG: Kyoto encyclopedia of genes and genomes
BMDMs: Bone marrow-derived macrophages
HUVECs: Human umbilical vein endothelial cells
5-aza: 5-Azacytidine
TGFβ: Transforming growth factor beta
