# Supplementary material for: Inactivation of pentraxin 3 suppresses M2-like macrophage activity and immunosuppression in colon cancer
Source: J Biomed Sci. 2024 Jan 20;31:10. doi: 10.1186/s12929-023-00991-7 (PMC10799366; doi:10.1186/s12929-023-00991-7)
Supplement: Supplementary file 1 — Additional file 1: Table S1. The primers for real-time polymerase chain reaction (real-time PCR). [file 12929_2023_991_MOESM1_ESM.docx]

**Additional file 1:**

**Table S1. The primers for real-time polymerase chain reaction (real-time PCR)**

| Gene | Product size | Species | Primer sequence (5’->3’) |
| --- | --- | --- | --- |
| PTX3 | 131 bp | Homo sapien | F: GGCCGAGAACTCGGATGATT  R: AGAGCTTGTCCCATTCCGAG |
| GAPDH | 186 bp | Homo sapien | F: CCCACTCCTCCACCTTTGAC R: TCTCTCTTCCTCTTGTGCTCTTG |
| ARG1 | 105 bp | Homo sapien | F: CTTGCGAGACGTAGACCCTG R: TCCATCACCTTGCCAATCCC |
| CD206 | 119 bp | Homo sapien | F: ATGGCTACTGGAAGACAGCAC R: GTGATCTGACTCCGGGCATC |
| VEGF | 148 bp | Homo sapien | F: TGTCTAATGCCCTGGAGCCT R: TTAACTCAAGCTGCCTCGCC |
| CD115 | 85 bp | Homo sapien | F: ATCGTCAACCTTCTGGGAGC R: AGTTGAGCAGGTCGCCATAG |
| IL10 | 135 bp | Homo sapien | F: CCTGCCTAACATGCTTCGAGA R: TGGCAACCCAGGTAACCCTT |
| Dectin-1 | 260 bp | Homo sapien | F: TGTGCTGCATCTCCTCCTTG R: TTAGGAGGACAAGGGCTGGA |
| CD163 | 72 bp | Homo sapien | F: TGGGCTAATTCCAGTGCAGG R: AGCTGACTCATTCCCACGAC |
| IL1B | 104 bp | Homo sapien | F: AGTACCTGAGCTCGCCAGTG R: CCTGGAAGGAGCACTTCATCTG |
| TNF | 77 bp | Homo sapien | F: GGAGAAGGGTGACCGACTCA R: AGTAGACCTGCCCAGACTCG |
| CXCL8 | 136 bp | Homo sapien | F: TACTCCAAACCTTTCCACCCC R: CCCAGTTTTCCTTGGGGTCC |
| CEBPB | 191 bp | Homo sapien | F: AGCGACGAGTACAAGATCCG R: AGCTGCTTGAACAAGTTCCG |
| IFNG | 84 bp | Homo sapien | F: CAAGTGATGGCTGAACTGTCG R: CTGGGATGCTCTTCGACCTC |
| Ptx3 | 143 bp | Mus musculus | F: TGCATTTGGGTCAAAGCCAC R: GCCAGCTTGTTCTCCTTTCC |
| Gapdh | 78 bp | Mus musculus | F: TGGTGAAGCAGGCATCTGAG R: TGAAGTCGCAGGAGACAACC |
| Cd206 | 117 bp | Mus musculus | F: AATCAGTGGTGGCCCTATGC R: GTGGATACTTGCCAGGTCCC |
| Arg1 | 1105 bp | Mus musculus | F: CTTGCGAGACGTAGACCCTG R: TCCATCACCTTGCCAATCCC |
