# Supplementary material for: Inactivation of pentraxin 3 suppresses M2-like macrophage activity and immunosuppression in colon cancer
Source: J Biomed Sci. 2024 Jan 20;31:10. doi: 10.1186/s12929-023-00991-7 (PMC10799366; doi:10.1186/s12929-023-00991-7)
Supplement: Supplementary file 2 — Additional file 2: Table S2. The primers for plasmid construction. [file 12929_2023_991_MOESM2_ESM.docx]

**Additional file 2:**

**Table S2. The primers for plasmid construction**

| Gene | Promotor region | Species | Primer sequence (5’->3’) |
| --- | --- | --- | --- |
| CEBPB | -889/+13 | Homo sapien | F: GAACACGAGCTCCCACTGACAGTAGCGACACC  R: CAGTATGCTAGCGCTCTGACTCGCTAAAGTTTCTC |
| VEGF | -881/-46 | Homo sapien | F: GAACACGGTACCCGCTCGGTGCTGGAATTTG R: CAGTATAGATCTTCGGCGAGCTACTCTTCCTC |
| ARG1 | -918/+30 | Homo sapien | F: GAACACGGTACCGCATAGAGGTTGACACCTTCCC R: CAGTATGCTAGCGAGCTCTCCAGTCAGTCAACC |
