# Supplementary material for: Inactivation of pentraxin 3 suppresses M2-like macrophage activity and immunosuppression in colon cancer
Source: J Biomed Sci. 2024 Jan 20;31:10. doi: 10.1186/s12929-023-00991-7 (PMC10799366; doi:10.1186/s12929-023-00991-7)
Supplement: Supplementary file 3 — Additional file 3: Figures. S1–S8. [file 12929_2023_991_MOESM3_ESM.pdf]

Additional information file 3: Supplementary Figures for

Inactivation of Pentraxin 3 Suppresses M2-like Macrophage Activity and Immunosuppression in Colon Cancer

Feng-Wei Chen[1], Yung-Ling Wu[2], Chao-Chun Cheng[1], Yu-Wei Hsiao[2], Jhih-Ying Chi[2], Liang-Yi Hung[2], Chih-Peng Chang[1,4], Ming-Derg Lai[3 *], Ju-Ming Wang[2, 5, 6, 7, 8 *]

Corresponds to Ju-Ming Wang (yumingw@mail.ncku.edu.tw)

This document includes:

Fig. S1 to S8

Supplementary Fig 1.

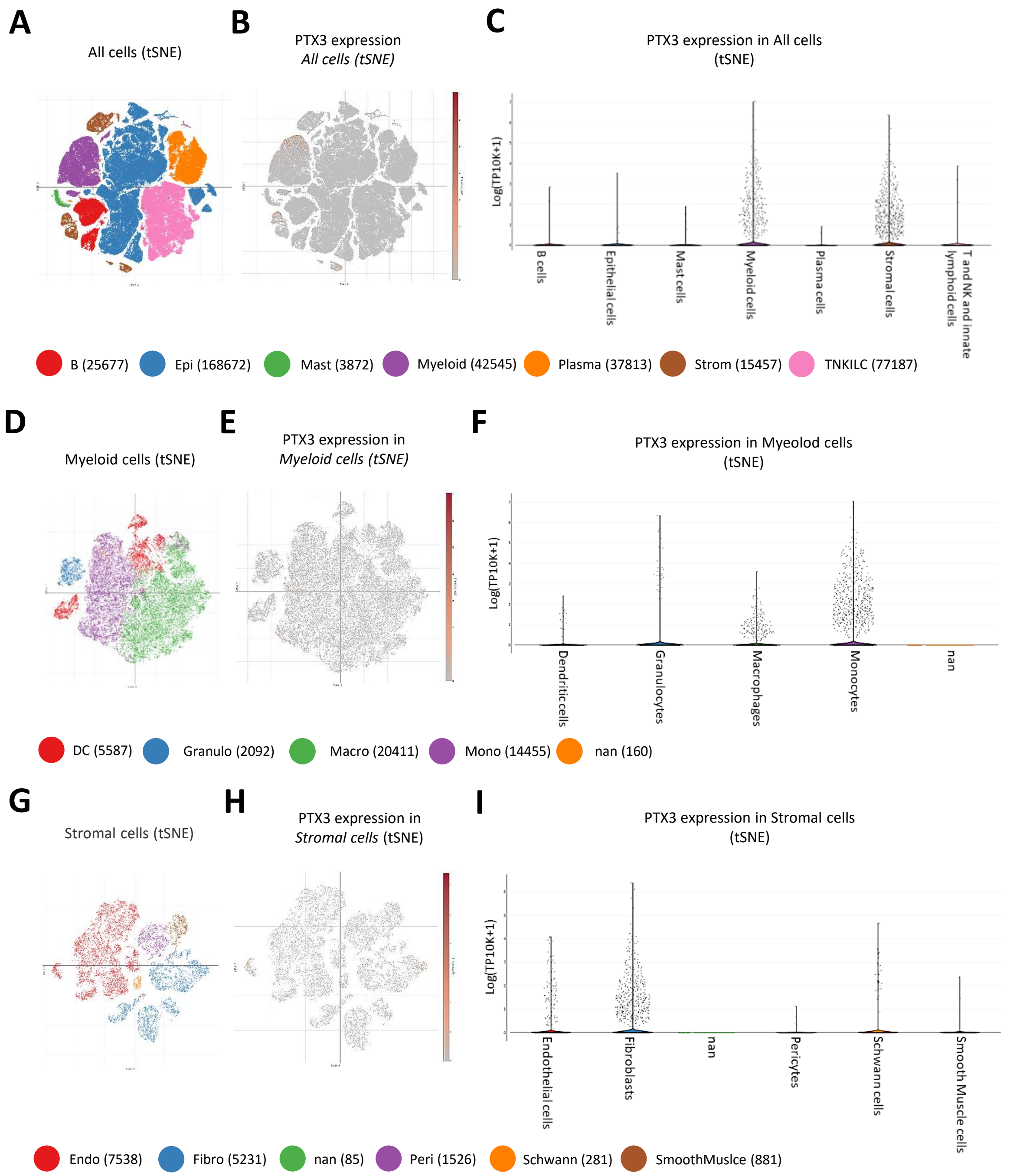

Additional file 1: Fig. S1 PTX3 is mainly expressed by stromal cells and myeloid cells in colon tumors. An open-access single-cell RNA-seq dataset from the Human Colon Cancer Atlas (c295) that provides transcriptional profiles of 371,223 cells from colorectal tumors and adjacent normal tissues. (A) The t-distributed scholastic neighbor embedding (t-SNE) plot of all cells shows 7 distinct clusters determined by cell type. (B) The t-SNE and (C) violin plots show PTX3 expression in 7 distinct clusters. (D) The t-distributed scholastic neighbor embedding (t-SNE) plot of myeloid cells shows 4 myeloid subclusters. (E) The t-SNE and (F) violin plots show PTX3 expression in the 4 myeloid subclusters. (G) The t-distributed scholastic neighbor embedding (t-SNE) plot of stromal cells shows 5 stromal subclusters. (H) The t-SNE and (I) violin plots show PTX3 expression in the 5 stromal subclusters. Nan stands for not-a-number, which means an undefined value.

Supplementary Fig 2.

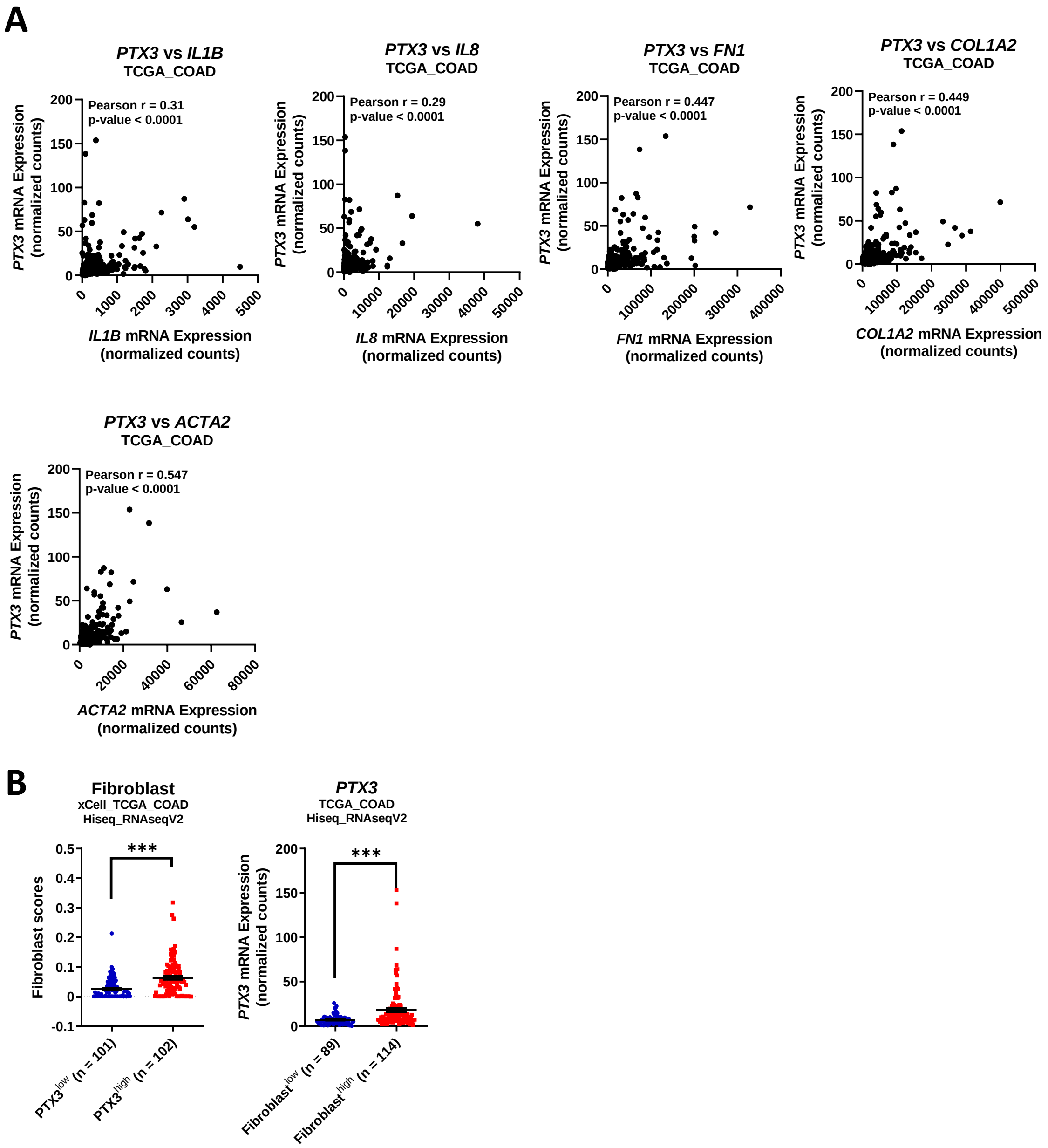

Additional file 1: Fig. S2 PTX3 expression is positively correlated with the expression of pro-inflammatory cytokine and stromal activation marker genes. (A) Pearson correlation analysis of *PTX3* with *IL1B*, *IL8*, *FN1*, *COL1A2*, and *ACTA2* in the TCGA COAD dataset (n = 287). (B) Fibroblast scores in 203 colorectal tumor samples from the TCGA dataset were extracted from the xCell database. The samples were divided into the PTX3^{high} (n = 102) and PTX3^{low} (n = 101) expression groups by the median cutoff value or the fibroblast^{high} (n = 114) and fibroblast^{low} (n = 89) score groups by the best cutoff value. P values were calculated by two-tailed unpaired Student's t test. *** represents a p value of < 0.001. Values on the plots are presented as the means ± SEMs.

Supplementary Fig 3.

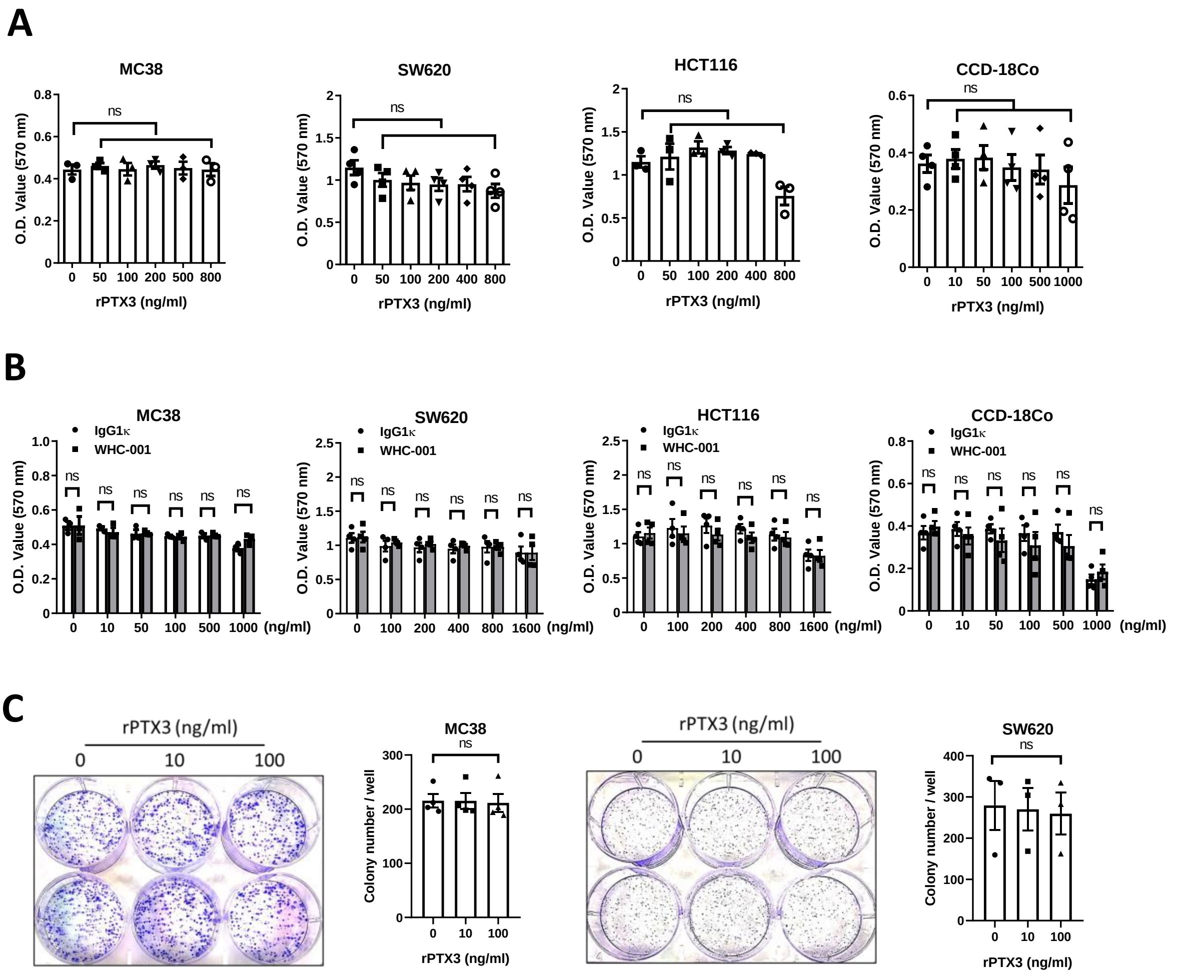

Additional file 1: Fig. S3 Exogenous PTX3 has no effect on cell proliferation and colony formation *in vitro*. (A, B) MTT assay of MC38, SW620, HCT116, and CCD-18Co cells treated with the indicated concentrations of recombinant PTX3 protein or antibody for 48 h. (C) Colony formation ability of MC38 (6 days) and SW620 (14 days) cells treated with recombinant PTX3 protein. P values were calculated by one-way ANOVA. ns represents nonsignificant difference. Values on the plots are presented as the means ± SEMs. Data are combined from 3 to 4 independent experiments.

Supplementary Fig 4.

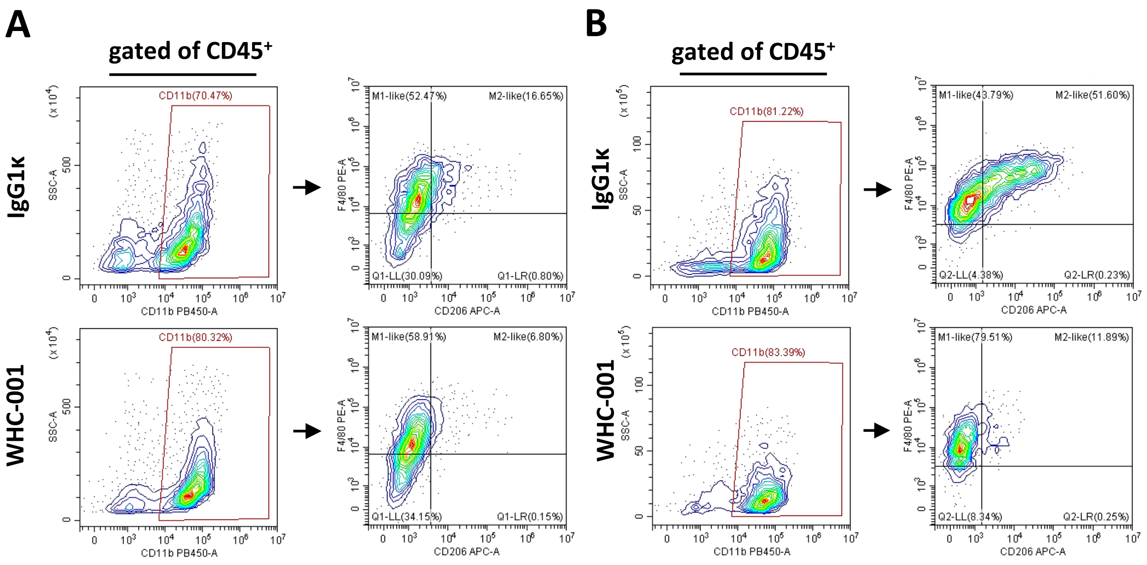

Additional file 1: Fig. S4 WHC-001 reduced the population of tumor-infiltrating M2-like macrophages. (A) The representative flow cytometry results of macrophages in Figure 4E and 4F. (B) The representative flow cytometry results of macrophages in Figure 4K and 4L. The percentage of each population was calculated as follows: M1-like macrophages (%M1-like × %CD11b) and M2-like macrophages (%M2-like × %CD11b).

Supplementary Fig 5.

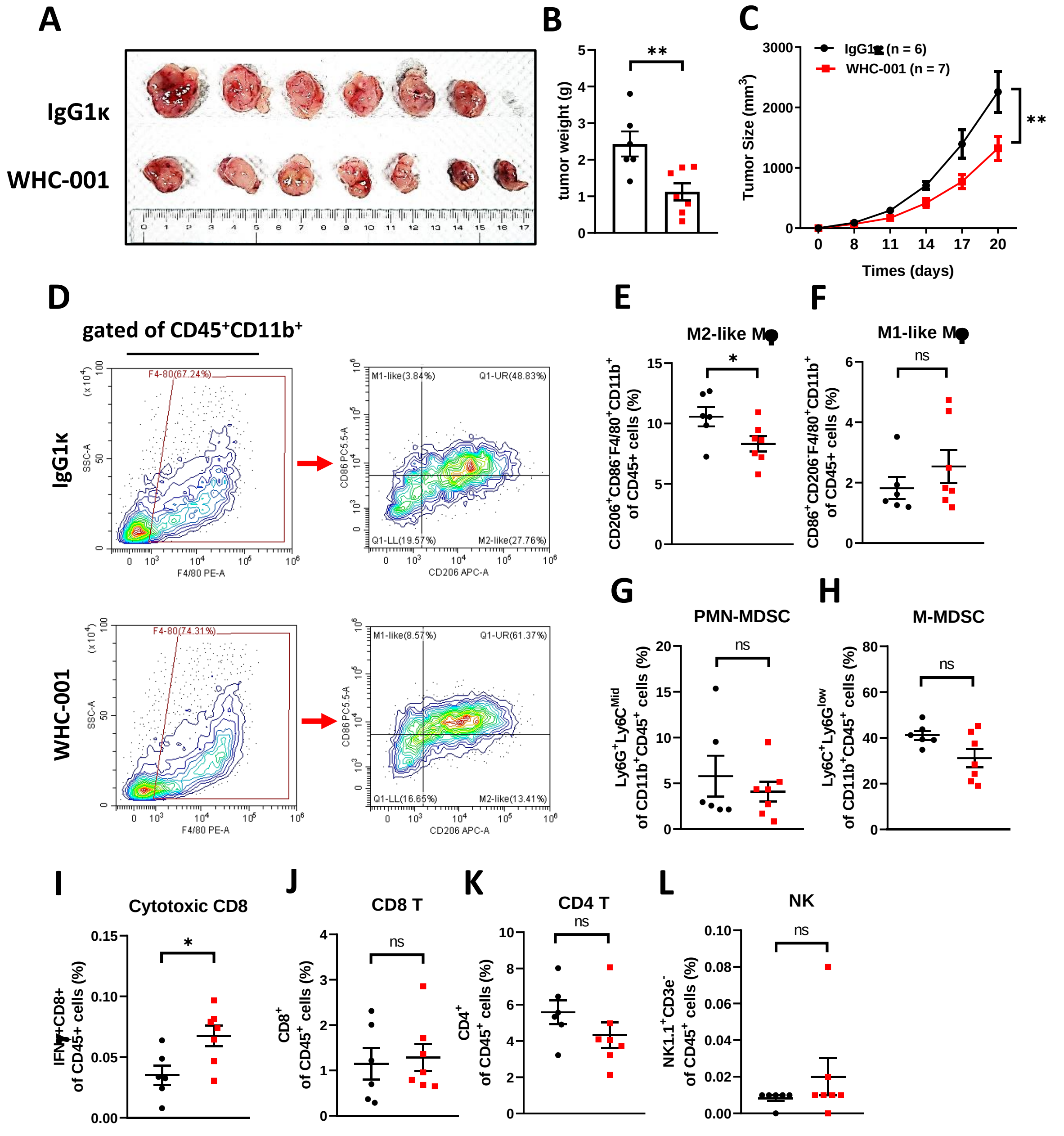

Additional file 1: Fig. S5 Blockade of PTX3 reduced tumor growth and the population of tumor-infiltrating M2-like macrophages and increased the population of cytotoxic $CD8^+$ T cells in the CT26 tumor model. (A-C) Illustration of tumors, tumor weights, and tumor growth curves in the CT26 subcutaneous model in BALB/c mice following i.p. administration of IgG1κ (n = 6) or WHC-001 (αPTX3 Ab, n = 7) (10 mg/kg) every 4 days for a total of 3 treatments. Tumor volume (mm^3) = ($L \times W^2$)/2. (D) The representative flow cytometry results of M1- and M2-like macrophages in tumors treated with IgG1κ or WHC-001. (E-L) Tumor-infiltrating immune populations were analyzed by flow cytometry. CD45 positive immune cells were gated for M2-like macrophages ($CD11b^+F4/80^+CD206^+CD86^-$), M1-like macrophages ($CD11b^+F4/80^+CD86^+CD206^-$), PMN-MDSCs ($Ly6G^+Ly6C^{Mid}/CD11b^+$), M-MDSCs ($Ly6C^+Ly6C^{low}/CD11b^+$), cytotoxic $CD8^+$ T cells ($IFN\gamma^+CD8^+$), total $CD8^+$ T cells ($CD8^+$), total $CD4^+$ T cells ($CD4^+$), and NK cells ($NK1.1^+CD3e^-$). The percentage of macrophage population was calculated as follows: M1-like macrophages (%M1-like × %F4/80 × %CD11b) and M2-like macrophages (%M2-like × %F4/80 × %CD11b). P values were calculated by two-tailed unpaired Student's t test, or two-way ANOVA. ns represents nonsignificant difference, * represents a p value of < 0.05, ** represents a p value of < 0.01, and *** represents a p value of < 0.001.

Supplementary Fig 6.

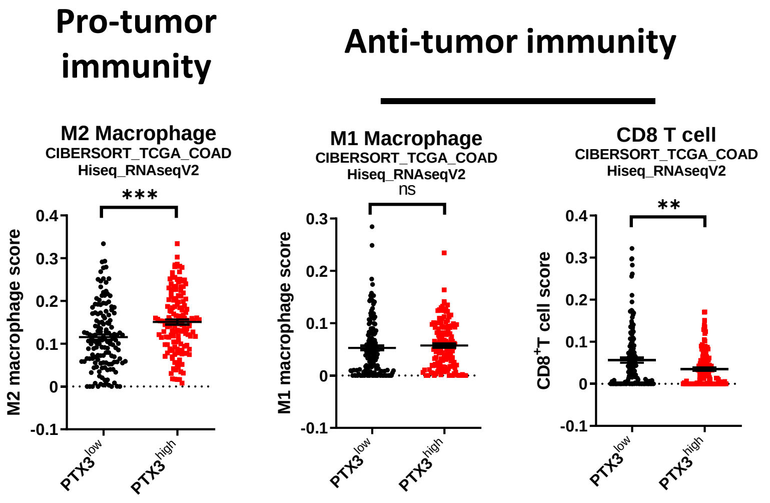

Additional file 1: Fig. S6 PTX3 expression is positively associated with M2 macrophage signature scores and negatively with CD8 T cells signature scores in the TCGA colon cancer dataset. Immune signatures (M2 macrophages, M1 macrophages, $CD8^+$ T cells) were estimated by using the CIBERSORT web-tool. Samples from the colon adenocarcinoma TCGA dataset were divided into the $PTX3^{high}$ (red, n = 144) and $PTX3^{low}$ (black, n = 143) groups by the median cutoff value. P values were calculated by two-tailed unpaired Student's t test. ns represents nonsignificant difference. Values on the plots are presented as the means ± SEMs. ns represents nonsignificant difference. ** represents a p value of < 0.01, *** represents a p value of < 0.001.

Supplementary Fig 7.

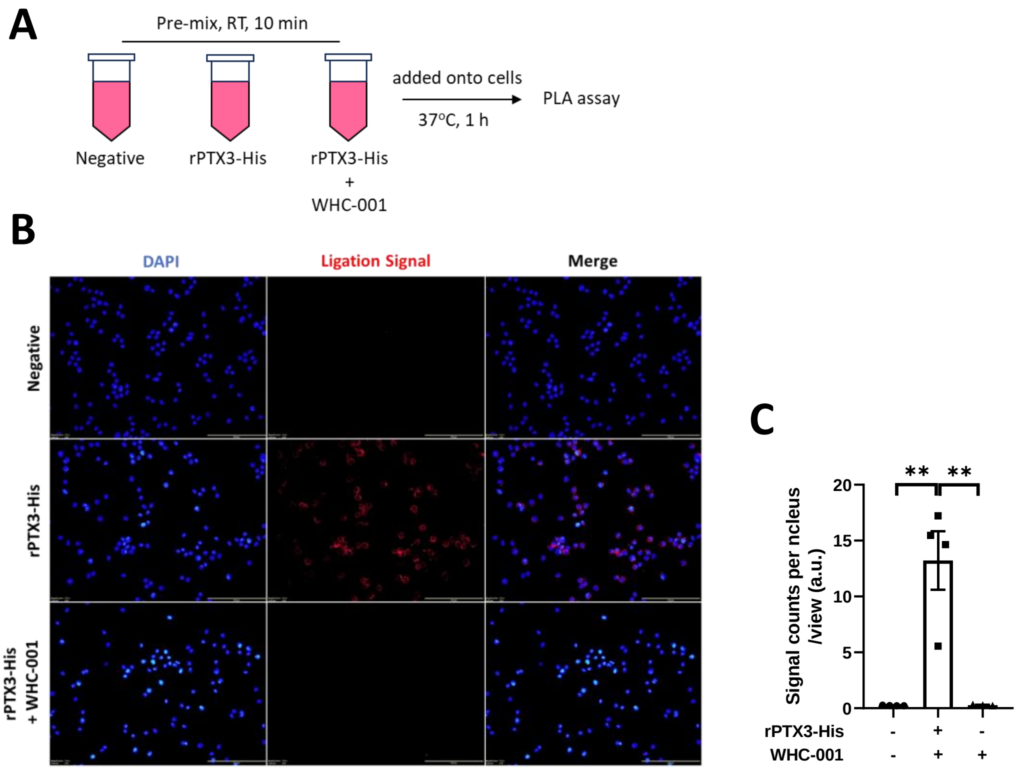

Additional file 1: Fig. S7 WHC-001 attenuates the binding of PTX3 to CD44 on THP-1-derived macrophages. (A) Illustration of the treatment protocol for the proximity ligation assay (PLA). (B) PLA was performed with both an anti-His-tag primary antibody and an anti-CD44 primary antibody, and the ligation signals were detected by fluorescence microscopy. The red signal indicates the interaction of PTX3-His and CD44, and the DAPI signal (blue) indicates the nucleus. The scale bar represents 200 μm. (C) The red signal count per nucleus in each view was quantified by ImageJ. P values were calculated by one-way ANOVA. ** represents a p value of < 0.01. Values on the plots are presented as the means ± SEMs. The data were analyzed in 4 random fields of each group.

Supplementary Fig 8.

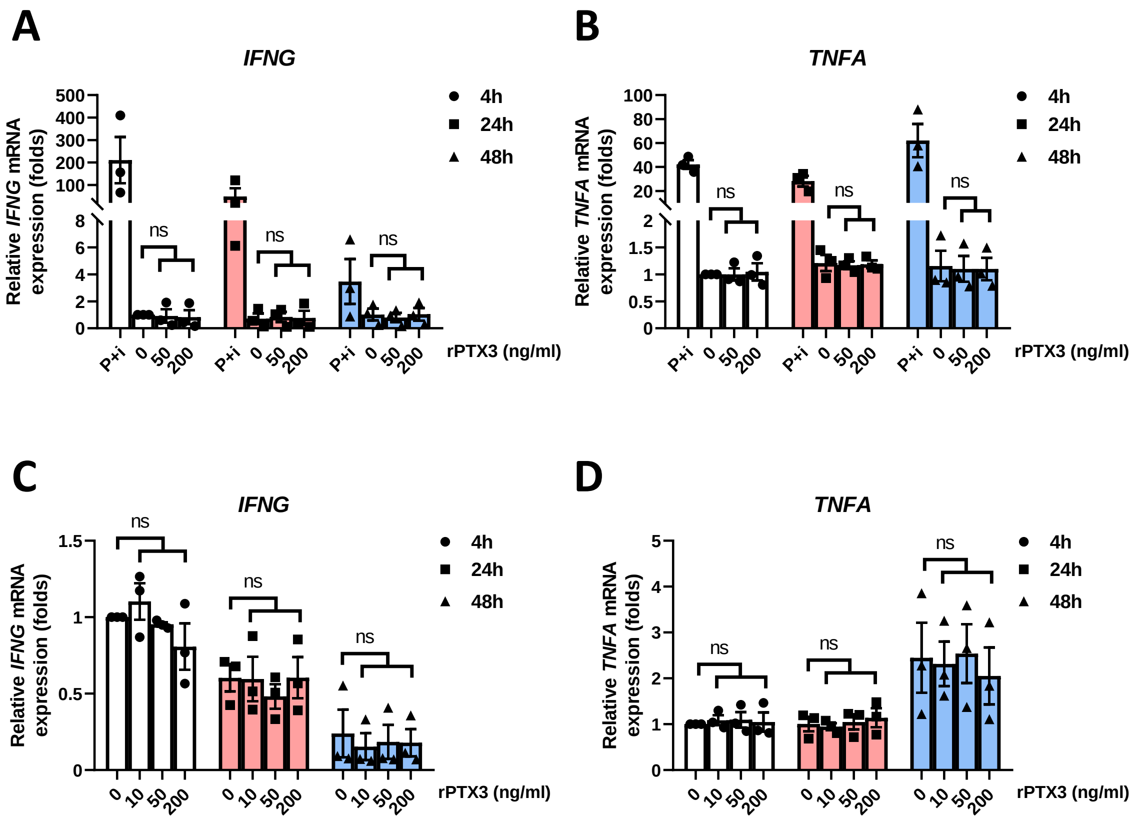

Additional file 1: Fig. S8 Exogenous PTX3 has no direct effect on regulating cytotoxic in Jurkat cells. (A, B) The mRNA expression of *IFNG* and *TNFA* in Jurkat cells treated with the indicated concentrations of rPTX3 for 4, 24, and 48 h. “P + i” represents stimulation with PMA and ionomycin. (C, D) The mRNA expression of *IFNG* and *TNFA* in Jurkat cells stimulated with PMA and ionomycin following treatment with the indicated concentrations of rhPTX3 for 4, 24, and 48 h. P values were calculated by one-way ANOVA. ns represents nonsignificant difference. Values on the plots are presented as the means ± SEMs. Data are combined from 3 independent experiments.
