# Supplementary material for: Inactivation of pentraxin 3 suppresses M2-like macrophage activity and immunosuppression in colon cancer
Source: J Biomed Sci. 2024 Jan 20;31:10. doi: 10.1186/s12929-023-00991-7 (PMC10799366; doi:10.1186/s12929-023-00991-7)
Supplement: Supplementary file 4 — Additional file 4: Additional materials and methods. [file 12929_2023_991_MOESM4_ESM.docx]

**Additional file 4:**

**Methods**

**Cell culture**

MC38, CT26, and HCT116 cells were kindly provided by Dr. LY Hung (National Cheng Kung University, Department of Biotechnology and Bioindustry Sciences, Taiwan, Tainan). HCT116, SW620, SW480, LoVo, HT29, Ls174T, and Colo205 cells were kindly provided by Dr. YL Chen (Chia Nan University of Pharmacy and Science, Taiwan, Tainan). Human colon fibroblasts (CCD-18Co; ATCC® CRL1459™), mouse embryonic fibroblasts (MEFs; MEFBL/6-1, ATCC® SCRC1008™, C57BL/6) and THP-1 cells (TIB-202™) were purchased from ATCC (Manassas, VA 20108, USA.). CT26, SW620, SW480, HT29, Ls174T, LoVo, and Colo205 cells and MEFs were maintained in Dulbecco’s modified Eagle’s medium (Life Technologies Co. Ltd., Grand Island, New York, United States; Gibco, #12900-082). HCT116 and THP-1 cells were maintained in RPMI-1640 medium (GE Healthcare Life Sciences, Logan, Utah, United States; HyClone, #SH30011.02). MC38 cells were maintained in DMEM supplemented with 0.1 mM nonessential amino acids, and 10 mM HEPES. CCD-18Co cells were maintained in Eagle's Minimum Essential Medium (Gibco, Life Technologies Limited Paisley, PA49RF, UK). All media were supplemented with 10% fetal bovine serum (Gibco, Life Technologies Corporation, 3175 Staley Rd., Grand Island, NY 14072, USA) (15% fetal bovine serum for MEFs) and 1X penicillin streptomycin solution (Corning, Mediatech, Inc., A Corning Subsidiary, 9345 Discovery Boulevard, Manassas, VA, 20109, USA). Cells were grown at 37 °C and in 5% CO_2_.

**MTT assay**

MC38 (1 × 10^3^), SW620 (4 × 10^3^), or HCT116 (3 × 10^3^) cells were seeded in 96-well plates; CCD-18Co (5 × 10^4^) cells were seeded in 24-well plates. After 24 h, the old media were replaced with 100 μl complete media containing the indicated concentrations of recombinant PTX3 protein or antibody (IgG1κ and WHC-001) for another 48 h. MTT solution (final concentration 0.5 mg/ml) (Sigma‒Aldrich, St. Louis, MO, USA) was added for another 2 h of incubation. Crystals are dissolved with DMSO and detected by measuring the O.D. at 570 nm.

**Angiogenesis assay**

For the precoating step, 100 μl Matrigel was poured into a 48-well plate for a 30 min incubation at 37°C. HUVECs (2.5 × 10^4^) were seeded into the Matrigel-coated 48-well plates with 100 μl of complete DMEM and 100 μl of THP-1-conditioned medium. After 18 h of incubation, the angiogenic ability was evaluated by determining the number of branch sites/nodes of tubes per field of view.

**Colony formation assay**

MC38 (0.5 × 10^3^) cells or SW620 (0.75 × 10^3^) cells were seeded in 6-well plates. After 24 h, the old media were replaced with 2 ml of complete media containing the indicated concentrations of recombinant murine or human PTX3 protein. For SW620 cells, recombinant PTX3 protein was refreshed on the 7^th^ day, and colonies were counted on the 14^th^ day. For MC38 cells, colonies were counted on the 6^th^ day. Colonies were stained with 0.2% crystal violet in 100% EtOH and colony numbers per well were determined.

**Reverse transcription polymerase chain reaction (RT‒PCR)**

Total RNA in lysates was extracted with TRIzol reagent (Invitrogen Life Technologies, Carlsbad, California, USA), chloroform and isopropanol. RNA (1-2 μg) was reverse transcribed into cDNA, which was diluted to 10-50 ng/μl for real-time PCR. Fast SYBR® Green Master Mix (Applied Biosystems, CA, USA) was used for real-time PCR. Relative expression was quantified by the 2^-ΔΔCT^ method. GADPH was used as an internal control. The primer sequences used for real-time PCR are listed in Supplementary Table 1.

**Enzyme-linked immunosorbent assay (ELISA)**

For assays with conditioned medium, cells were grown in 6-cm dishes with 2 ml of complete medium. When the cell confluence reached 80%, the old medium was replaced with 2 ml of serum-free medium. After another 24 h of incubation, the supernatants were obtained by centrifugation at 3000 rpm for 10 min to remove cell debris. For assays with plasma, mice were anesthetized, and cardiac puncture was performed to collect blood into a 26G syringe containing 100 μl heparin. Plasma was centrifuged at 4000 xg for 15 min at 4°C, and the supernatant was harvested. The Human Pentraxin 3 ELISA Kit (#E20031001, Leadgene Biochemical, Inc) and Mouse Pentraxin 3/TSG-14 Quantikine ELISA Kit (#MPTX30, R&D System) were used. All assays were performed according to the manufacturer's protocol.

**THP-1 cells differentiation**

7 × 10^5^ THP-1 cells were seeded in 6-cm dishes or 6-well plate with 2 ml of complete medium overnight. M0 macrophage differentiation was induced by treatment with 320 nM PMA for 6 h (THP-1 macrophage, M0). Floating cells were washed off with PBS and the adherent cells were treated with 20ng/ml IL-13 and 20 ng/ml IL-4 for 18 h (M2 macrophage) or 50 ng/ml rhPTX3 for 18 h.

**Collection of conditioned medium**

For ELISA, cells were seeded in 6-cm dishes, and when the cell confluence reached 80-90%, the medium was replaced with 2 ml serum-free medium for 48 h, and the supernatants were collected for ELISA. For collection of THP-1 macrophage conditioned medium, THP-1 macrophages stimulated with the indicated cytokines were washed with PBS and incubated with 2 ml serum-free medium for 20 h, and the medium was collected and centrifuged at 3000 rpm for 5 min to remove cell debris. Furthermore, in the angiogenesis assay, THP-1 macrophage conditioned medium was concentrated at 16-fold; in Jurkat cell activation assay, THP-1 macrophage conditioned medium was concentrated at 20-fold by using an Amicon® Ultra4 Centrifugal Filter Unit (7500 xg, 15 min, 4°C, angle rotator).

**Jurkat cell activation**

For conditioned medium treatment, 5 × 10^4^ Jurkat cells in 1 ml of complete RPMI-1640 medium were seeded in a 12-well plate and incubated overnight. The cells were treated with 100 μl of concentrated THP-1 macrophage conditioned medium (20-fold concentration) and treated with or without stimulators (20 ng/ml PMA+1.33 μM ionomycin+1x Golgi Stop) for 4 h and the cell lysates were harvested for RNA extraction. For PTX3 treatment, 1-20 × 10^5^ Jurkat cells in 1 ml of complete RPMI-1640 medium were seeded in 12-well plates, incubated overnight and stimulated with or without PMA + ionomycin following treatment with or without the indicated concentrations of hPTX3 for 4, 24, and 48 h.

**Luciferase reporter constructs and luciferase reporter assay**

The promoters of each gene (CEBPB, VEGF, ARG1) were inserted into the PGL3 basic vector. The primers for amplification of each gene are listed in Supplementary Table 2. Plasmids were transfected into HEK293 cells by using TransIT-X2 Reagent (Mirus Bio LLC) according to the manufacturer’s instructions. After 48 h, the cells were lysed with 200 μl of lysis buffer (25 mM Tris-HCl, 25 mM K-phosphate, 4 mM EGTA, 1% Triton X-100, 10% glycerol, 2 mM DTT) and centrifuged for 15 min at 13,000 rpm. The supernatants were collected and mixed with 350 μl luciferase assay buffer (25 mM Tris-HCl, 25 mM K-phosphate, 20 mM MgSO_4_, 4 mM EGTA, 2 mM ATP, 1 mM DTT) and 50 μl luciferin. The mixture was vortexed at max. speed for 10 sec and luciferase activity was measured by GloMax® 20/20 Luminometer (Promega) immediately.

**Western blot assays**

Cell lysates were harvested with RIPA lysis buffer (150 mM NaCl; 1% NP-40; 0.5% sodium deoxycholate; 0.1% SDS; 25 mM Tris; pH 7.4) supplemented with protease inhibitors (10 ng/ml aprotinin, 10 ng/ml leupeptin, 0.58 mM PMSF, 1 mM DTT, 1 mM Na_3_VO_4_, 0.5 nM NaF). Protein concentrations were measured by using Bio-Rad Protein Assay Dye Reagent Concentrate (Bio-Rad Laboratories Inc., 1000 Alfred Nobel Drive, Hercules, CA, 94547, USA). Total protein lysates (15-30 μg of total protein) were mixed with 4X protein loading dye (40 mM Tris-HCl, 40% glycerol, 1.6% SDS, 4% β-ME, 0.08% bromophenol blue) and boiled at 95 °C. Proteins in the samples were separated by 10% SDS‒PAGE (Separating gel - 10% acrylamide; 0.375 M Tris-HCl; pH 8.8; 0.1% SDS; 0.1% ammonium persulfate; 0.0004% TEMED; stacking gel – 5.1% acrylamide; 0.125 M Tris-HCl; pH 6.8; 0.1% SDS; 0.1% ammonium persulfate; 0.001% TEMED). The separated proteins were transferred onto a 100% methanol-activated PVDF membrane, which was then incubated in blocking buffer (5% w/v nonfat dry milk in 1X TBST) at room temperature on a shaker for 1 hour. After being washed with 1X TBST, the membrane was incubated with specific primary antibodies at 4 °C overnight. Primary antibodies against PTX3 (sc-373951, Santa Cruz), GAPDH (GTX100118, GeneTex), phospho-CREB1 (#9191S, Cell Signaling), CREB1 (sc-204, Santa Cruz), and CEBPB (sc-150, Santa Cruz) were used. After the membrane was washed with 1X TBST, secondary antibodies (HRP-conjugated anti-mouse, GTX213111-01, or anti-rabbit IgG, GTX213110-01) were hybridized to the primary antibodies at room temperature on a shaker for 1 hour. After being washed with 1X TBST, the membrane was rinsed with Trident plus Western HRP Substrate (GTX400006, GeneTex) to induce the enzyme-substrate reaction, and the chemiluminescence signals were detected by a ChemiDoc^TM^ Imaging System (Bio-Rad). Images were analyzed by Bio-Rad CFX Manager 2.1 software (Bio-Rad).
